# Genome-wide study of *Cerrena unicolor* 87613 laccase gene family and their mode prediction in association with substrate oxidation

**DOI:** 10.1186/s12864-023-09606-9

**Published:** 2023-08-30

**Authors:** Long-Bin Zhang, Wu-Wei-Jie Yang, Ting-Ting Qiu

**Affiliations:** 1 Fujian Key Laboratory of Marine Enzyme Engineering, Fuzhou University, Fuzhou, 350116 Fujian China; 2 College of Biological Science and Engineering, Fuzhou University, Fuzhou, 350116 Fujian China

**Keywords:** *Cerrena unicolor*, Genome, Laccase, Gene family, Transcriptional response, 3D structure, Molecular docking

## Abstract

**Background:**

Laccases are green biocatalysts with wide industrial applications. The study of efficient and specific laccase producers remains a priority. *Cerrena* species have been shown to be promising basidiomycete candidates for laccase production. Although two sets of *Cerrena* genome data have been publicly published, no comprehensive bioinformatics study of laccase gene family in *C. unicolor* has been reported, particularly concerning the analysis of their three-dimensional (3D) structures and molecular docking to substrates, like ABTS and aflatoxin B_1_ (AFB_1_).

**Results:**

In this study, we conducted a comprehensive genome-wide analysis of laccase gene family in *C. unicolor* 87613. We identified eighteen laccase genes (*CuLac*s) and classified them into three clades using phylogenetic analysis. We characterized these laccases, including their location in contig 5,6,9,12,15,19,26,27, gene structures of different exon-intron arrangements, molecular weight ranging from 47.89 to 141.41 kDa, acidic pI value, 5–15 conserved protein motifs, signaling peptide of extracellular secretion (harbored by 13 *CuLac*s) and others. In addition, the analysis of *cis*-acting element in laccase promoters indicated that the transcription response of *CuLac* gene family was regulatable and complex under different environmental cues. Furthermore, analysis of transcription pattern revealed that *CuLac8*, *12* and *CuLac2*, *13* were the predominant laccases in response to copper ions or oxidative stress, respectively. Finally, we focused on the 3D structure analysis of CuLac proteins. Seven laccases with extra transmembrane domains or special sequences were particularly interesting. Predicted structures of each CuLac protein with or without these extra sequences showed altered interacting amino acid residues and binding sites, leading to varied affinities to both ABTS and AFB_1_. As far as we know, it is the first time to discuss the influence of the extra sequence on laccase’s affinity to substrates.

**Conclusions:**

Our findings provide robust genetic data for a better understanding of the laccase gene family in *C. unicolor* 87613, and create a foundation for the molecular redesign of CuLac proteins to enhance their industrial applications.

**Supplementary Information:**

The online version contains supplementary material available at 10.1186/s12864-023-09606-9.

## Introduction

Laccase (EC 1.10.3.2) is a kind of multicopper-containing oxidase that is capable of oxidizing both phenolic and non-phenolic substrates, while simultaneously reducing molecular oxygen to water [1]. Laccase is found in various organisms, including plants, bacteria, fungi and even insects. Notably, the properties of laccase vary depending on the source from which it is derived. Fungal laccases, particularly those produced by white-rot species, generally exhibit much higher redox potentials than those from other sources [2, 3]. This increased redox potential provides greater redox capability and allows for a broader substrate spectrum [4, 5]. As a result, fungal laccase has garnered significant interest as a potential tool for industrial biotechnology applications, such as lignin degradation, decolorization, detoxification and polymeric synthesis [6]. Additionally, fungal laccase plays a vital role in the biological process of fruiting body formation, spore pigmentation, stress response, and pathogenesis [2, 7]. Therefore, it is crucial to comprehensively study fungal laccases to understand the properties of their origins better and to facilitate their application in various industries.

The white-rot fungus *Cerrena unicolor* is highly valued for its medicinal properties, which shows healing effects towards various human ailments [8, 9]. Meanwhile, *Cerrena* species are of particular interest in the field of laccase applications due to its high yield of laccase [3]. Previous researches have primarily focused on characterizing and applying single laccase, such as Lcc3 in *Cerrena* sp. WR1 [10], Lac1 in *Cerrena* sp. HYB07 [11], and Lac2 in *C. unicolor* 6884 [12]. However, it has been discovered that laccases in basidiomycetes are encoded by a gene family consisting of 4–12 members [13–16]. The largest laccase gene family discovered to date is 17 members found in *Coprinopsis cinerea* [17]. With the advent of next-generation sequencing technology, it is now possible to obtain complete versions of laccase gene families from transcriptomic or genomic data. Consequently, scientists have shifted their focus from single genes to multigene family studies [18]. To date, two *Cerrena* species transcriptomes and two *Cerrena* species genome sequences have been publicly published. The transcriptomic data from *C. unicolor* 6884 and *C. unicolor* FCL139 have unveiled 13 and 8 laccases [19, 20], respectively. However, gene transcription can be influenced by the presence or absence of various inducers, which might result in the omission of inducible laccases. Therefore, genome-wide sequencing has become a successful method to avoid inducer interference and completely detect the laccase gene family [21]. Currently, the entire genome of *C. unicolor* 303 has been annotated by JGI (https://mycocosm.jgi.doe.gov/Cerun2/Cerun2.home.html, accessed on 5 May 2023) without any additional study [22]. Furthermore, *C. unicolor* SP02 genome has been released in the NCBI database (accession No. PRJNA704632), revealing several genes involved in lignocellulose degradation [23]. However, there is still a critical knowledge gap concerning *Cerrena* laccase gene family, particularly with regard to their 3D structure prediction. It is also worth mentioning that eight laccases cloned from *C. unicolor* HYB07 share 47–93% sequences identities with 10 annotated laccases in the genome of *C. unicolor* 303 [24]. In addition, *C. unicolor* SP02 harbors 8 annotated laccases in the genome [23]. These results indicate that laccase gene families and genome sequences might differ significantly between *Cerrena* species. Therefore, the study of a novel *Cerrena* species genome is necessary for expanding genetic information within the genus *Cerrena* and comprehensively elucidating the properties and 3D structure of their laccase gene family.

In the present study, we obtained a strain *C. unicolor* 87613, which has shown promise in producing extracellular laccases with a maximal activity of 415.66 U/mL after 6-day fermentation. In comparison, other *Cerrena* species displayed lower laccase activities ranging from 121.7 U/mL to 333.2 U/mL after shake-flask incubation of 5, 12 or 14 days [12, 25, 26], respectively. This makes *C. unicolor* 87613 an advantageous resource due to its ability to produce laccases at earlier and higher levels. Therefore, Exploring the laccase gene family in *C. unicolor* 87613 is of great value. To further understand the potential of *C. unicolor* 87613, we carried out experiments to investigate its laccase gene family (Additional file 2: Fig. S1). Through the utilization of Next Generation Sequencing, we obtained an overview of the *C. unicolor* 87613 genome. Within this genome, we identified a laccase gene family comprising 18 members, designated as *CuLac1*–Cu*Lac18*. By analyzing both their nucleic acid and amino acid sequences, we were able to determine their properties and expression patterns. Additionally, we constructed three-dimensional (3D) structure models of each laccase protein. Furthermore, we selected two representative substrates for laccase molecular docking experiments: 1) 2,2’-azino-bis-(3-ethylbenzothiazoline-6-sulfonic acid) (ABTS), which is commonly used in laccase activity assay [27, 28], and 2) Aflatoxin B_1_ (AFB_1_), a well-known toxic human carcinogen in moldy grain products, which could be efficiently degraded by laccase [12, 29]. By conducting molecular docking studies of laccase with ABTS and AFB_1_, we aimed to gain a better understanding of laccase function and its potential applications. Our results provide valuable insights into the laccase gene family in *C. unicolor* 87613 and lay a foundation for better production and commercialization of *Cerrena* laccases.

## Methods

### Microbial strains and culture conditions

Strain *Cerrena unicolor* 87613 was obtained from China Forestry Culture Collection Center (CFCC) and stored at the Key Laboratory of Marine Enzyme Engineering of Fujian Province, Fuzhou University. The strain was rejuvenated on Potato Dextrose Agar (PDA solid media with 2% glucose and 1.5% agar), followed by static incubation at 30 ℃ for about 4–5 days. For submerged fermentation of extracellular laccase, the strain was incubated in PDA liquid media, shaking at 200 rpm and 30 ℃.

### Oxidative activity assay of extracellular and intracellular laccases

Strain *C. unicolor* 87613 was obtained from China Forestry Culture Collection Center (CFCC). We performed oxidative activity assays of extracellular and intracellular laccases over a 12-day fermentation at 30 ℃ and 150 rpm at daily intervals. 1 mL of fermentation supernatant or 0.1 g of partial cultures were collected, followed by the procedure outlined in a previous study [24]. One unit (U) of enzyme oxidative activity was defined as the amount of enzyme required to oxidize 1 µmol of ABTS per min. All measurements were carried out in triplicate.

### Extraction and sequencing of genome in ***C.unicolor*** 87613

To extract the integrative genome from *C. unicolor* 87613 cultures, we employed the cetyltrimethylammonium bromide (CTAB) method. We dissolved the precipitated genome in water and used the NEBNext®Ultra™ DNA Library Prep Kit (NEB, USA) for library construction. Novogene company (Beijing, China) performed library preparation and high-throughput sequencing, using Illumina NovaSeq PE150. The reliability of our sequencing results was demonstrated in Additional file 1: Table S1.

### Genome assembly and genome component prediction

The genome assembly was performed by Novogene company (Beijing, China) using SOAP denovo software. Briefly, the obtained valid data (clean data) were used. Different k-mers (default selections 95, 107, 119) were selected for genome assembly. Based on the optimal k-mer, we adjusted the parameters (-d -u -R - F, etc.) to obtain preliminary assembly results. The gap-closing software was used to fill the gap in preliminary assembly results. It was also used to remove the same lane pollution by filtering the reads with low sequencing depth (less than 0.35 of the average depth) to obtain the final assembly result. Finally, fragments below 500 bp were filtered out, and the result was counted for gene prediction.

Genome component prediction included the prediction of the coding gene, repetitive sequences and non-coding RNA. We used the Augustus 2.7 program to proceed with ab initio gene finding. Protein homology detection and intron resolution were performed with the GeneWise software and the uniref90 non-redundant protein database. Subsequently, we aligned the known expressed sequence tags (ESTs) and full-length cDNAs to the genome, followed by PASA alignment assemblies. We further used the EVidenceModeler (EVM) to compute weighted consensus gene structure annotations. Then, we used PASA to update the EVM consensus predictions and add both UTR annotations and models for alternatively spliced isoforms. The interspersed repetitive sequences were predicted using the Repeat Masker (http://www.repeatmasker.org/). The tandem Repeats were analyzed by the TRF (Tandem repeats finder). Transfer RNA (tRNA) genes were predicted by the tRNAscan-SE. Ribosome RNA (rRNA) genes were analyzed by the rRNAmmer. sRNA, snRNA and miRNA were predicted by BLAST against the Rfam database. All parameters required in each server were set as the default values.

### Analysis of gene function

Gene functions were predicted by two major databases, including Gene Ontology (GO, http://www.geneontology.org) [30] and Kyoto Encyclopedia of Genes and Genomes (KEGG, http://www.genome.jp/kegg) [31]. A whole genome Blast search (E-value less than 1e^− 5^, minimal alignment length percentage larger than 40%) was performed against the above databases. Genes were functionally classified into three categories: biological process (BP), cellular component (CC) and molecular function (MF) by GO analysis, and enriched into four kinds of KEGG pathways: metabolism, genetic information processing, environmental information processing and cellular processes. All parameters required in each analyzing website were set as the default values.

### Genome-wide identification of laccase family genes in ***C.unicolor*** 87613

The achieved clean data were assembled and used for prediction and functional annotation of genes. According to the database of Pfam, Swiss-Prot, and Carbohydrate-Active enzymes (CAZy), the candidate laccase sequences were selected. All candidate laccase sequences were further verified according to the conserved laccase domains (Cu-oxidase: PF00394, Cu-oxidase_2: PF07731, Cu-oxidase_3: PF07732), using the HAMMER database (www.ebi.ac.uk/Tools/hmmer/search/hmmscan, 30 December 2022).

### Multiple sequence alignment and phylogenetic analyses

The amino acid sequences of putative laccases from *C. unicolor* 87613 (CuLac) were aligned by Clustalw. The phylogenetic tree was then constructed with the neighbor-joining (NJ) method (1000 bootstrap replicates) in MEGA 7.0. In addition, the copper-binding region (L1–L4) and the substrate binding loops in the amino acid sequence of each putative *CuLac* were recognized according to previous reported [32].

### Determination of physical localization and gene structure of CuLac genes

Contigs indicated the units for sequence assembly of *C. unicolor* 87613 genomes. Based on the genomic analyses, the contig length and the relatively physical localization of eighteen *CuLac* genes were determined. The exon-intron arrangement of each putative *CuLac* gene was analyzed and performed by Gene Structure Display Server (GSDS v2.0, http://gsds.cbi.pku.edu.cn/, 9 August 2022). All parameters required in the server were set as the default values.

### Analyses of conserved motif, signal peptide, secreted pattern, glycosylation of laccase proteins

The amino acid sequence of all putative *CuLac*s was used to determine their conserved motifs (Additional file 1: Table S2) by the MEME server v.5.4.1 (http://meme-suite.org/tools/meme, 30 December 2022). The signal peptides and corresponding cleavage sites in each laccase were predicted by SignalP 5.0 online server [33]. Notably, the predicted signal peptide was only acceptable as its predicted probability was higher than 0.50. The transmembrane domain prediction was performed by using DeepTMHMM server (https://dtu.biolib.com/DeepTMHMM). The glycosylation sites of putative *CuLac*s were predicted by NetNGlyc server 1.0 for N-linked glycosylation [34]. All parameters required in each online server were set as the default values.

### Identification of ***cis***-acting elements

The upstream regions (1.5 kb) from the initial site (ATG) of each putative *CuLac* gene were selected to detect cis-acting elements. Ten CRRs with their conserved sequences were used (Additional file 1: Table S3).

### RNA extraction and qRT-PCR assays

*C. unicolor* 87613 was routinely grown on PDA media with 2% (w/v) glucose, 1.5% (w/v) peptone and 100 µM CuSO_4_ (Control group). Besides, strains were also grown on PDA media with different stimuli: (1) substituted fructose for glucose; (2) reduced the amount of peptone to 0.15% (w/v); (3) added the amount of Cu^2+^ to 250 µM; (4) supplied with 5 mM H_2_O_2_ or (5) supplied with 1% (w/v) sodium lignosulfonate (SL) at fermentation day 4. After incubation at 30 ℃, 200 rpm for total 6 days, each culture was collected for total RNA extraction under the action of an RNAisoTM Plus Reagent (TaKaRa, Dalian, China). The extracted RNAs were reversely transcribed into cDNA using a PrimeScript RT reagent kit (TaKaRa). Three cDNA samples (standardized by dilution) derived from each of the three independent cultures were used as templates to quantify transcripts of eighteen laccases via qPCR with paired primers (Additional file 1: Table S4), using the fungal 18 S rRNA as an internal standard. A threshold cycle (2^−ΔΔCT^) method was used to calculate relative transcription level of each gene in strains treated with different supplies. Specifically, the cDNA sequences of *CuLac17*, *18* were too similar to be separately measured. Hence, the transcriptional levels of *CuLac17*, *18* were simultaneously detected using the same paired primers. The transcript level of laccase genes in strains without any treatment was used as a standard.

### Homology modeling of each laccase and their molecular docking to ABTS or AFB_1_

3D structure models of each CuLac were constructed by homology modeling in Discovery Studio (DS) 2019 (V19.1.0., Accelrys Software Inc., San Diego, CA, USA) and I-TASSER (https://seq2fun.dcmb.med.umich.edu//I-TASSER/) [35]. A model of one CuLac with the highest identity and similarity was selected for further studies. The protonation state of the protein and the orientation of the hydrogen atoms were optimized at pH 7.0.

The 3D structure of ABTS and AFB_1_ were obtained from PubChem (https://pubchem.ncbi.nlm.nih.gov/) with Compound CID being 9570474 and 14403, respectively. The molecular docking simulation of each CuLac model with ABTS or AFB_1_ was performed by DS CDOCKER. The 2D diagram was used to predict their Receptor-Ligand interactions. The binding model with the highest value of -CDOCKER_ENERGY was selected from different conformers for each ligand.

## Results

### Determination of early and high laccase production in ***C. unicolor*** 87613

**Fig. 1:**
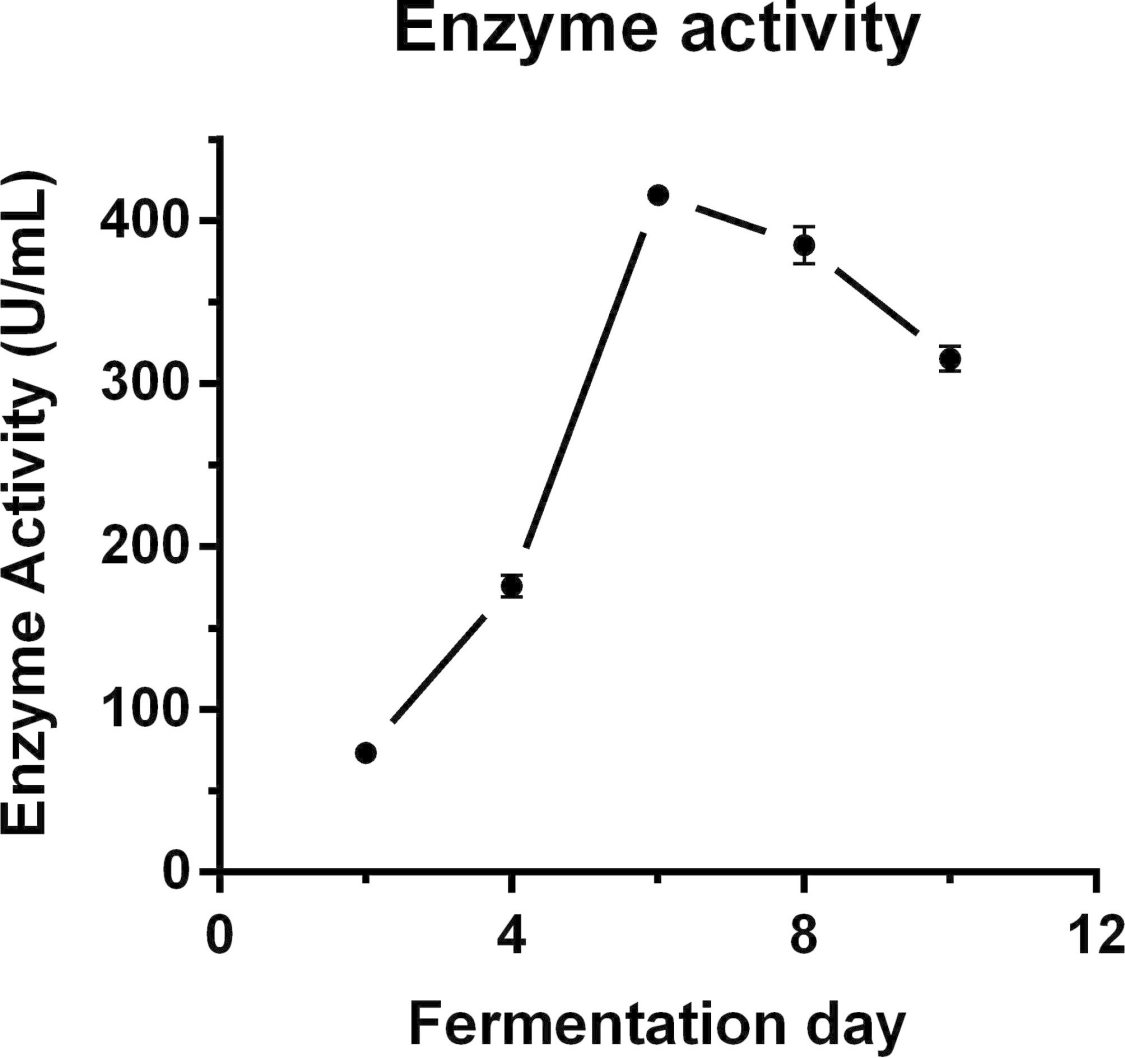
Laccase productivity of *C. unicolor* 87613 with early and high yielding rate. The highest extracellular laccase activity was up to 415.66 U/mL after 6-day fermentation

*C. unicolor* 87613, obtained from strain resource library CFCC, had been previously confirmed to belong to Cerrena genus and was found to have high extracellular laccase (exLac) activity when tested from fermentation day 2–10 (Fd2–10) using ABTS. The estimated laccase activity was 73.33 U/mL at Fd2, and reached the peak value of 415.66 U/mL at Fd6 (Fig. 1). As compared to other *Cerrena* species with maximal exLac activity of 121.7–333.2 U/mL after fermentation of 5–14 day [12, 25, 26], C. unicolor 87613 could be defined as both early- and high-laccase-production fungus. These high levels of *C. unicolor* 87613 laccase production at an early stage gain the strain desirable attention.

### General genome characteristics of ***C. unicolor*** 87613

To gain a better understanding of this promising fungus, the genomic version of *C. unicolor* 87613 was obtained through next-generation sequencing. The analysis revealed that the complete genome size was 40.11 Mb with a GC content of 46.64% (Additional file 1: Table S1). After the reads containing low-quality bases (mass value ≤ 20) over a certain percentage (the default was 40%) were removed, genome assembly were performed by SOAP denovo software. As anaylzing the assembly (83.10% completeness) data, the genome consisted of 56 contigs, with the longest contig length at 4.44 Mb and the shortest at 4.82 Kb (Additional file 2: Fig. S2A). In total, 26,799 gene sequences were predicted, with 12,515 annotated as protein-coding genes. Further analysis revealed that 6,209 genes were assigned to three major categories: biological process (BP), molecular function (MF) and cellular component (CC) based on the Gene Ontology (GO) database (Additional file 2: Fig. S2B). In the BP category, the majority of genes (3,233) were involved in “metabolic process”, while the majority of genes (3,425) in MF were associated with “binding” function. The CC category had 1,575 genes responsible for forming a “cell”. Additionally, 7,210 genes were annotated in the KEGG database (Additional file 2: Fig. S2C). The most enriched category, “metabolism”, contained 12 subcategories, including “carbohydrate metabolism”, “amino acid metabolism”, “lipid metabolism”, and so on. These results draw a much clearer genetic background of *C. unicolor* 87613.

### Identification of laccase gene family in ***C. unicolor*** 87613

*C. unicolor* 87613 showed advantages in laccase productivity. A genome-wide analysis was carried out to further investigate the genetic basis of its laccase production. Eighteen targeted genes were identified with three conserved laccase domains (Cu-oxidase: PF00394, Cu-oxidase_2: PF07731, Cu-oxidase_3: PF07732). These 18 candidates (named *CuLac1*–*18*) were categorized as members of laccase gene family (Table 1). The targeted genes encoded laccase isozymes consisting of 430–1,304 amino acids (AAL), and their molecular weight (MW) ranged from 47.89 kDa to 141.41 kDa. Their theoretical isoelectric point (pI) ranged from 4.62 to 6.20. Analysis of SignalP and TMHMM2.0 revealed that most laccase isozymes (*CuLac1*–*CuLac16*) were predicted to have signal peptides, and 5 isozymes (*CuLac2*, *8*, *16*, *17*, *18*) contained one or more transmembrane domains (TDs). These results suggest that most laccases are secretory proteins, except for the five isozymes that are transmembrane proteins. All laccases showed multiple N-glycosylation sites except for *CuLac5*.

**Table 1:** The predicted and tallied physiochemical properties of 18 putative laccase genes in *C. unicolor* 87613

Gene name	Sequence ID	AAL (aa)	MW (kDa)	pI	Sig. Pep. cleavage site	TD	Local.	N-Glyc
*CuLac1*	A08365	526	56.75	4.98	SYA-AI	0	Extr	4
*CuLac2*	A01510	764	82.58	5.17	SYA-AI	3	PM	4
*CuLac3*	A01716	430	47.89	5.36	NWA-DG	0	Extr	3
*CuLac4*	A07743	1,304	141.41	6.20	AFA-AI	0	Extr	6
*CuLac5*	A08371	526	56.88	4.95	TYA-GI	0	Extr	0
*CuLac6*	A00710	815	89.93	6.06	VNA-AI	0	Extr/Nucl	8
*CuLac7*	A00709	510	55.03	4.62	VSA-AI	0	Extr	7
*CuLac8*	A10074	844	90.46	4.85	ANA-AI	1	PM	10
*CuLac9*	A04285	483	52.11	5.73	AFG-AI	0	Extr	2
*CuLac10*	A01646	516	55.58	4.88	TYA-AI	0	Extr	4
*CuLac11*	A07755	516	56.16	5.17	AYA-VL	0	Extr	7
*CuLac12*	A02259	518	55.94	5.88	AFA-AI	0	Extr	2
*CuLac13*	A08431	516	55.18	4.94	VFA-AI	0	Extr	2
*CuLac14*	A08410	537	57.48	4.62	AFA-AV	0	Extr	2
*CuLac15*	A07756	516	55.20	4.78	AFA-AI	0	Extr	2
*CuLac16*	A08247	627	68.56	4.75	VLA-GV	1	PM	12
*CuLac17*	A04359	597	65.14	5.56	NONE	1	PM	13
*CuLac18*	A08888	612	66.79	5.17	NONE	1	PM	14

AAL is an abbreviation to amino acid length; MW is abbreviated for molecular weight; pI is stand for theoretical isoelectric point; Sig. Pep. is shorted for signal peptide; TD is an abbreviation to transmembrane domain; Local. is shorted for localization status; N-Glyc is shorted for N-linked glycosylation

The distribution of the 18 laccase genes was mapped onto the *C. unicolor* 87613 contigs using their respective starting positions (Additional file 2: Fig. S3). Contig 5 contained the largest number of *CuLac* genes. Interestingly, most laccase genes were located at the tops or middle parts of the contigs, with the exception of *CuLac16* and *CuLac18* located at the bottoms of the contigs. *CuLac1*, *4*, *5*, *11*, *15* were densely located together at the top of contig 5, while *CuLac8*, *9*, *12*, *17* were individually in contig 9, 19, 26, 27, respectively.

### Analysis of phylogenetic relationships, gene structures, and conserved motifs in ***CuLac*** gene family members

Given the fact that the genome data of *Cerrena* sp. 303 have been publicly released [22], the phylogenetic tree was constructed based on *CuLac1*–*CuLac18* and other laccases in *Cerrena* sp. 303 (Additional file 2: Fig. S4). Three distinct clades were observed: Clade I contained *CuLac1*–*7* and *Lac4*, *6*, *7*, *9* in Ce*rrena* sp. 303; Clade II consisted of *CuLac8*–*15* and Ce*rrena* sp. 303 *Lac1*, *2*, *3*, *5*, *8*; and Clade III included *CuLac16*–*18*, which exhibited low protein homology with the other laccase genes. *CuLac2*, *3*, *4*, *6*, *7*, *16*, *17*, *18* were identified as novel laccases, as their identity and/or similarity were less than 70% compared to those in *Cerrena* sp. 303 (Additional file 2: Fig. S5).

**Fig. 2:**
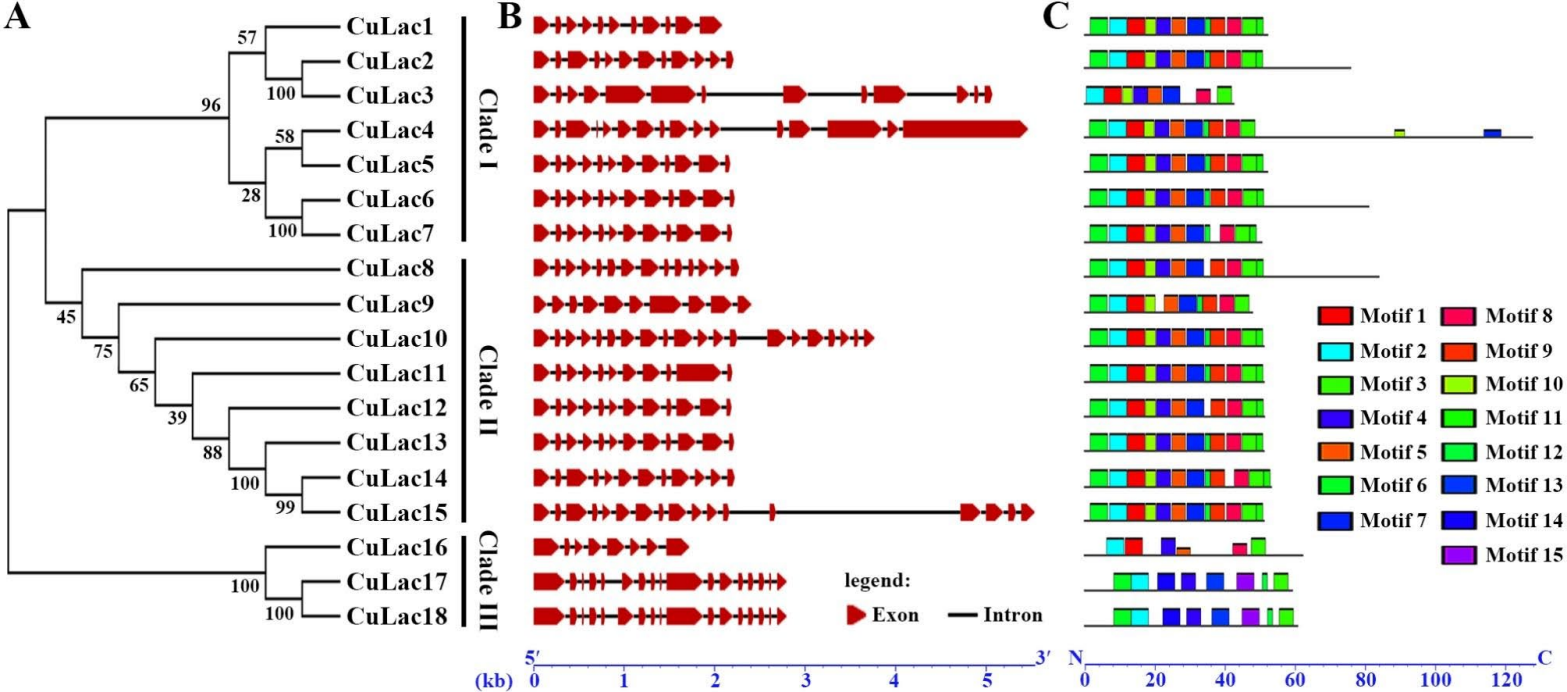
Structural analysis of laccase family from *C. unicolor* 87613. **(A)** A neighbor-joining tree of 18 *CuLac* genes constructed using MEGA 7.0. **(B)** The exon-intron structure of *CuLac* genes. Red boxes represented exons; black lines indicate introns. **(C)** The motif patterns of CuLac proteins. The colored square represents 15 conserved motifs, shown in Additional file 1: Table S2. Both gene length and protein length can be estimated using the scale at the bottom

In the simplified phylogenetic tree, *CuLac1*–*7* in Clade I exhibited low homology with each other, while *CuLac8*–*15* in Clade II displayed high homology (Fig. 2A, Additional file 2: Fig. S5). *CuLac16*–*18* in Clade III showed significant differences from the other CuLac isozymes. These results indicate that there might be variations in the structure and properties within the *CuLac* gene family. This was initially investigated by studying their gene structures. The number of introns in *CuLac* genes ranged from 7 to 19 (Fig. 2B). Noteworthily, not all *CuLac* genes within the same clade had similar exon-intron structures. For instance, the exon-intron arrangements significantly differed between *CuLac3*, *4* and their paralogous genes in Clade I. Similarly, different exon-intron arrangements were observed for *CuLac10*, *15* in Clade II and *CuLac16* in Clade III. These results indicate that *CuLac* gene structures exhibit sequence diversity even within the same clades.

Using the MEME program, we subsequently identified fifteen conserved motifs in the amino acid sequences of each CuLac (Fig. 2C, Additional file 1: Table S2). Despite differences in gene structure, most CuLacs contained the same twelve motifs arranged in a similar pattern. However, some exceptions were noted, such as CuLac2, 4, 6, 8, which had extended C-terminal sequences, and CuLac3, 16, 17, 18, which had altered motifs. These exceptions in each clade might suggest their unique properties and/or functions.

### Analysis of multiple sequence alignment

**Fig. 3:**
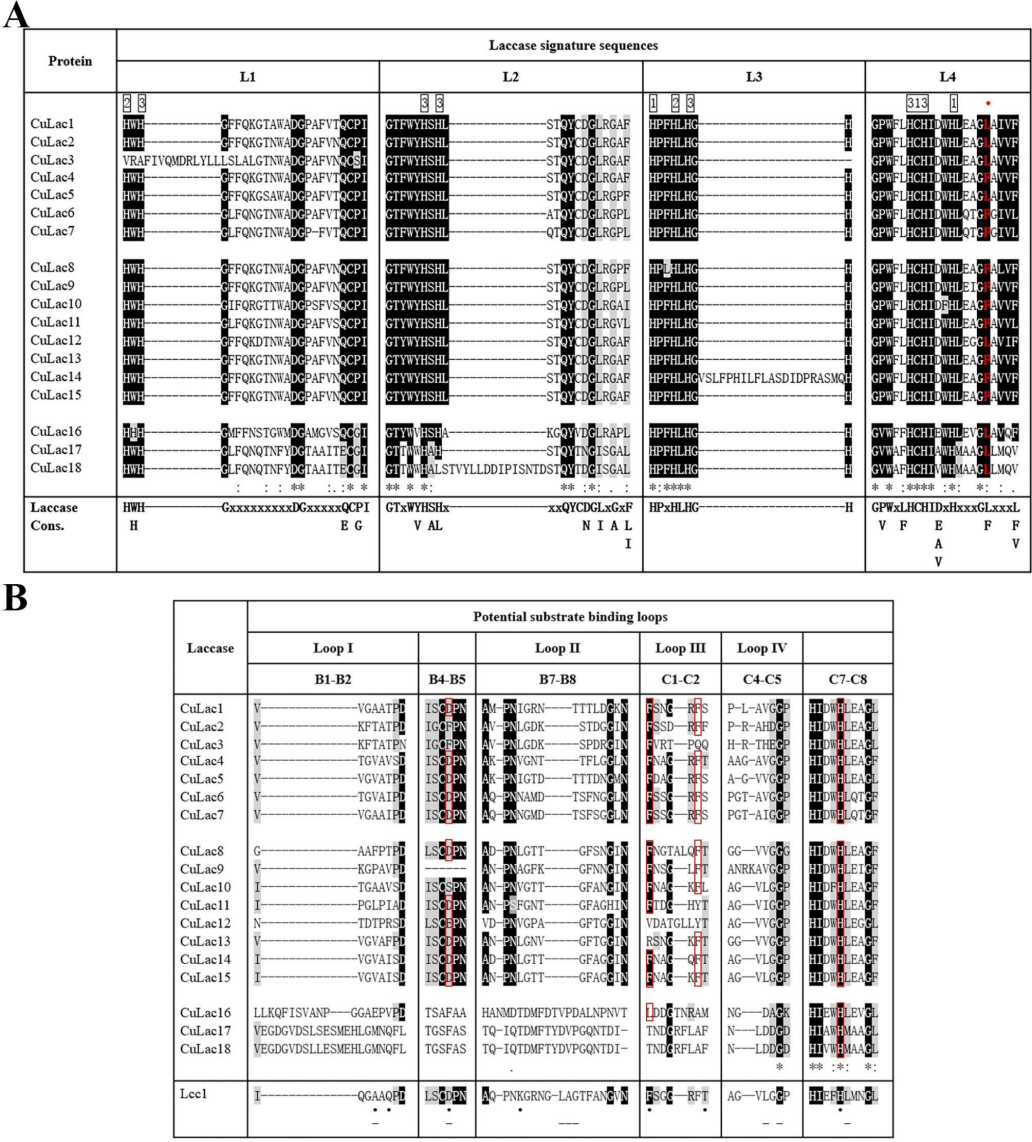
Analysis of 18 CuLac amino acid sequences. **(A)** The sequence alignments of four laccase signature sequences (L1–L4). The histidine **(H)** and cysteine **(C)** residues involved in copper binding are numbered according to the copper type (1, 2, and 3 for type1, type 2, and type 3, respectively) they bind. **(B)** The sequence alignments of the potential substrate binding loops of 18 CuLac proteins and the laccase (Lcc1) in *Coprinopsis cinerea* [17]

The alignment of amino acid sequences for *CuLac* gene family is presented in Fig. 3. The laccase signature sequences (L1–L4) in the amino acid sequences of each CuLac were analyzed following the report by Kumar [32]. Of the 18 CuLacs, 16 contained complete laccase signature sequences, which included ten conserved histidine residues and one cysteine residue (Fig. 3A). Two histidine residues were absent in L1 of CuLac3, and a histidine-to-leucine change occurred in L2 of CuLac18. In the active site of laccase, a copper cluster center consisting of one T1-Cu, one T2-Cu and two T3-Cu was in charge of electron transferring from substrates to oxygen molecules [36, 37]. The absent histidine residues in CuLac3 and CuLac18 are known to interact with T2-Cu or T3-Cu [32], indicating that CuLac3 and CuLac18 might have unstable copper cluster centers. Notably, a cysteine to valine or threonine change occurred in L2 of CuLac16 or CuLac17, 18, respectively. Additional amino acid sequences were found in L1 of CuLac3, L2 of CuLac18 and L3 of CuLac14, which might affect their interaction with copper cluster center. Moreover, the alignments showed that each CuLac had either leucine or phenylalanine at the tenth amino acid residue after cysteine in L4 (Fig. 3A). Evidence had proved that leucine or phenylalanine at this position always contributed to an impressive high redox potential of laccase’s type I copper [38, 39]. Therefore, we defined all CuLac isozymes as valuable laccases with considerably high redox potential.

All 18 CuLacs possess substrate binding loops (Loop I–IV in Fig. 3B), as described from 3D structure analysis of crystallized laccases [40–42]. According to the previous reports [13, 40], their protein-ligand interaction residues in the pocket formed by the substrate binding loops were examined. As a result, CuLac1, 4–8, 14, 15 had more substrate binding sites than others, while CuLac17 and CuLac18 had the fewest binding sites (Fig. 3B). The analysis of substrate binding loops further supports that CuLacs within the same phylogenetic clade exhibit characteristic and functional diversities.

### ***Cis***-regulatory region predicted in ***CuLac*** promoter sequence

**Fig. 4:**
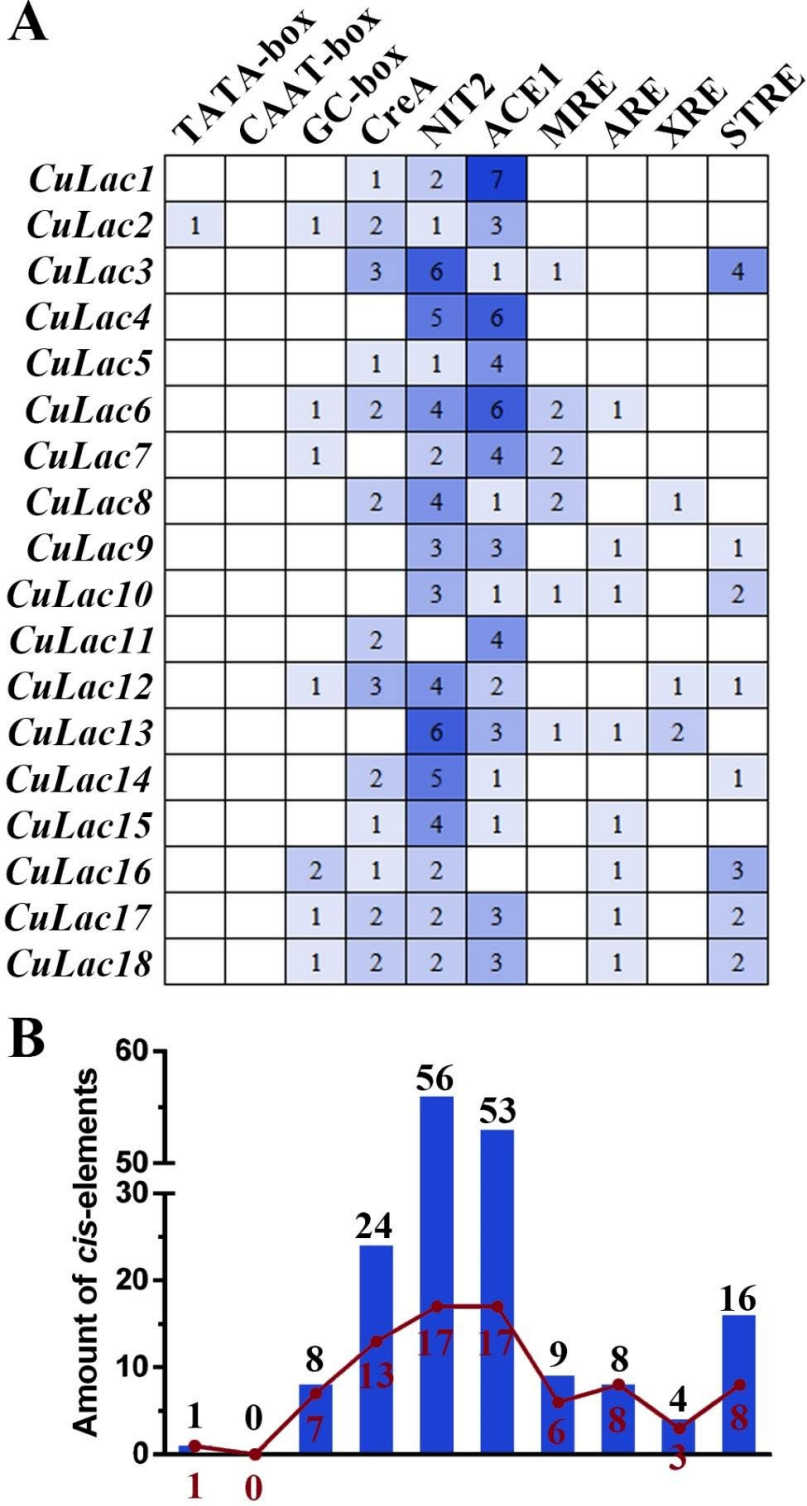
The kind and the number of *cis*-acting elements in the promoter regions of 18 *CuLac* genes. (**A**) The presentation of 10 targeted *cis*-acting elements in each CuLac promoter. (**B**) The number of *cis*-acting elements in the CuLac promoters. The red line represents the number of genes containing the corresponding *cis*-acting elements in promoter regions, and the boxes indicate the total number of each targeted *cis*-acting element in CuLac promoters

To investigate the regulation of *CuLac* gene expression, we analyzed ten conserved sequences (Additional file 1: Table S3) as cis-acting elements in each *CuLac* promoter region (~ 1,500 bp). *CuLac2* was the only gene to harbor the TATA-box (Fig. 4A). However, other *cis*-acting elements were present in each promoter region of *CuLac* gene family, including GC-box, nutrient-responsive elements (CreA [43] and NIT2 [44]), ion-responsive elements (ACE1 [45] and MRE [46]) and environment-responsive elements (ARE, XRE [47] and STRE [27]). Among those, NIT2 was the most frequently occurring element in the *CuLac* gene family (up to 56 times), whereas XRE elements were the least common (Fig. 4B). Interestingly, 17 of 18 *CuLac* genes had NIT2 and ACE1 elements, while only three *CuLac* genes contained XRE elements (Fig. 4). The variety of *cis*-acting elements in *CuLac* gene family implicates that each *CuLac* gene might respond differently to environmental factors, such as carbon and nitrogen sources, metal ions and aromatic compounds.

### Transcriptional response of ***CuLac*** gene family to different agencies

**Fig. 5:**
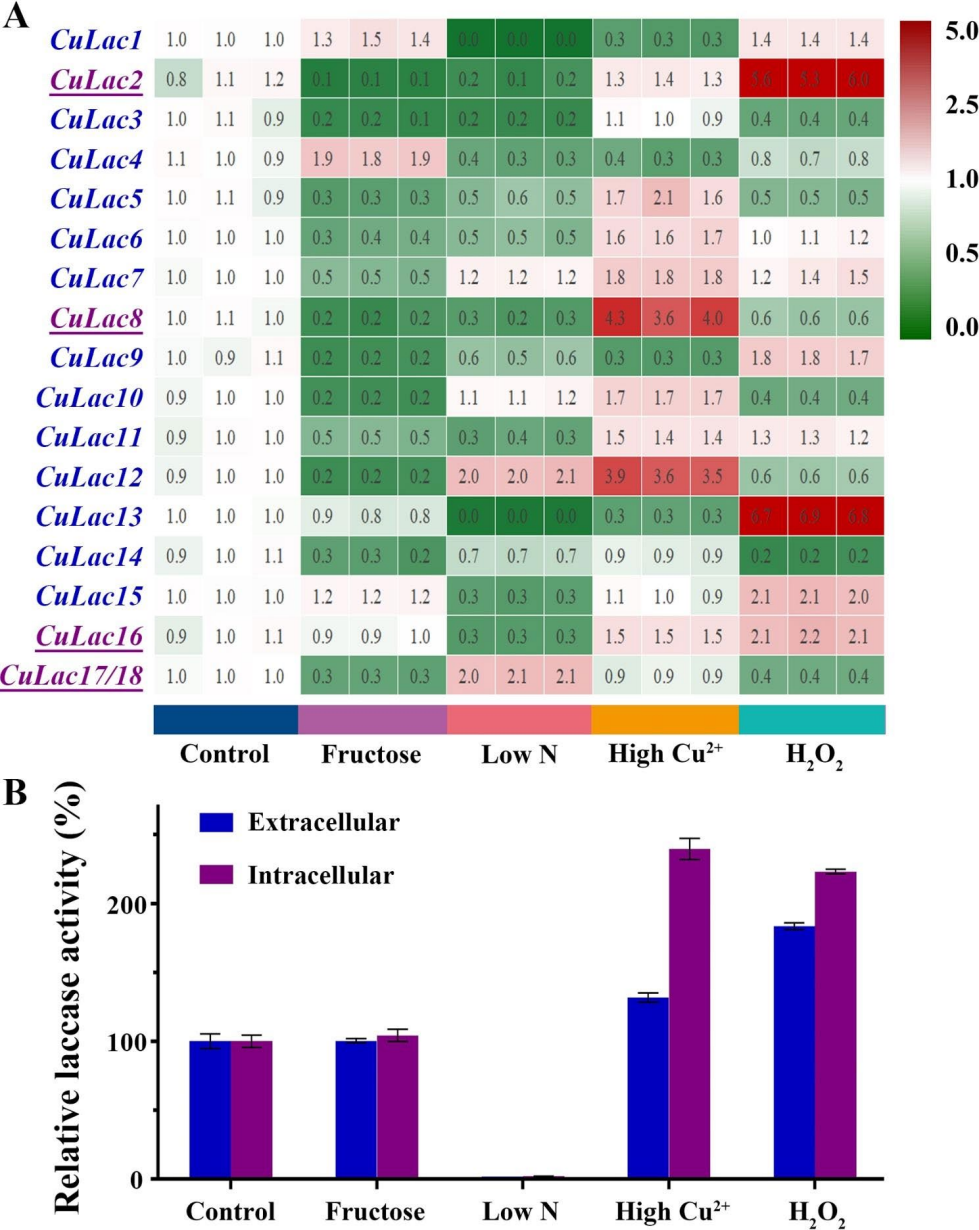
The alteration of transcription level and enzyme activity of CuLac family in response to different stimuli. **(A)** The transcription response of each *CuLac* gene under different fermentation conditions supplied with fructose (2%, w/v), low concentration of peptone (0.15%, w/v), high concentration of copper ion (250 µM) or H_2_O_2_ (5 mM) as compared to the control group (supported with 2% (w/v) glucose, 1.5% (w/v) peptone and 100 µM CuSO_4_). **(B)** The oxidation activities of both crude extracellular and intracellular CuLac after 6-day fermentation under different conditions above

Transcription of most laccase genes is inducible towards varied environmental stimuli. We measured the transcription changes of each *CuLac* gene in *C. unicolor* 87613 grown under different conditions. Compared to the control group, the substitution of glucose with fructose significantly repressed the transcription level of most *CuLac* genes, while *CuLac1*, *4*, *15* were slightly increased (Fig. 5A). Its corresponding extracellular and intracellular laccase (ExLac and InLac) activities were both similar to those of the control group (Fig. 5B). Notably, our previous analysis predicted that *CuLac2*, *8*, *16*, *17*, *18* encoded transmembrane proteins (Table 1), suggesting their contributive roles in InLac activity. Meanwhile, the rest are secretory proteins contributing to ExLac activity. Therefore, under the condition of fructose fermentation, retaining transcriptional levels of secretory *CuLac1*, *4*, *15* might maintain ExLac activity at the control level, as well as *CuLac16* contributing to InLac activity. Under the less-nitrogen condition, *CuLac12*, *17*/*18* transcription levels were upregulated, but the rest were generally downregulated, resulting in a drastic reduction of crude laccase activities (about 98% of both ExLac and InLac activities as compared to those under the control condition) (Fig. 5B). High concentration of Cu^2+^ specifically stimulated the transcription of secretory *CuLac5*, *6*, *7*, *10*, *11*, *12* and transmembrane *CuLac2*, *8*, *16*, which resulted in the increase of ExLac activity (by 31%) and InLac activity (by 139%) (Fig. 5A,B). Supporting H_2_O_2_ also boosted the transcription of secretory *CuLac1*, *7*, *9*, *11*, *13*, *15* and transmembrane *CuLac2*, *16* (Fig. 5A), leading to an increase in both ExLac and InLac activities by 83% and 123%, respectively (Fig. 5B). These altered transcription patterns of the *CuLac* gene family suggest that each *CuLac* gene is individually regulated by different stimuli and responds in different ways. The integrative action of all *CuLac* gene family members finally determined the crude extracellular and intracellular laccase activities.

### 3D structure modeling of CuLac isozymes and their molecular docking to ABTS and/or AFB_1_

**Fig. 6:**
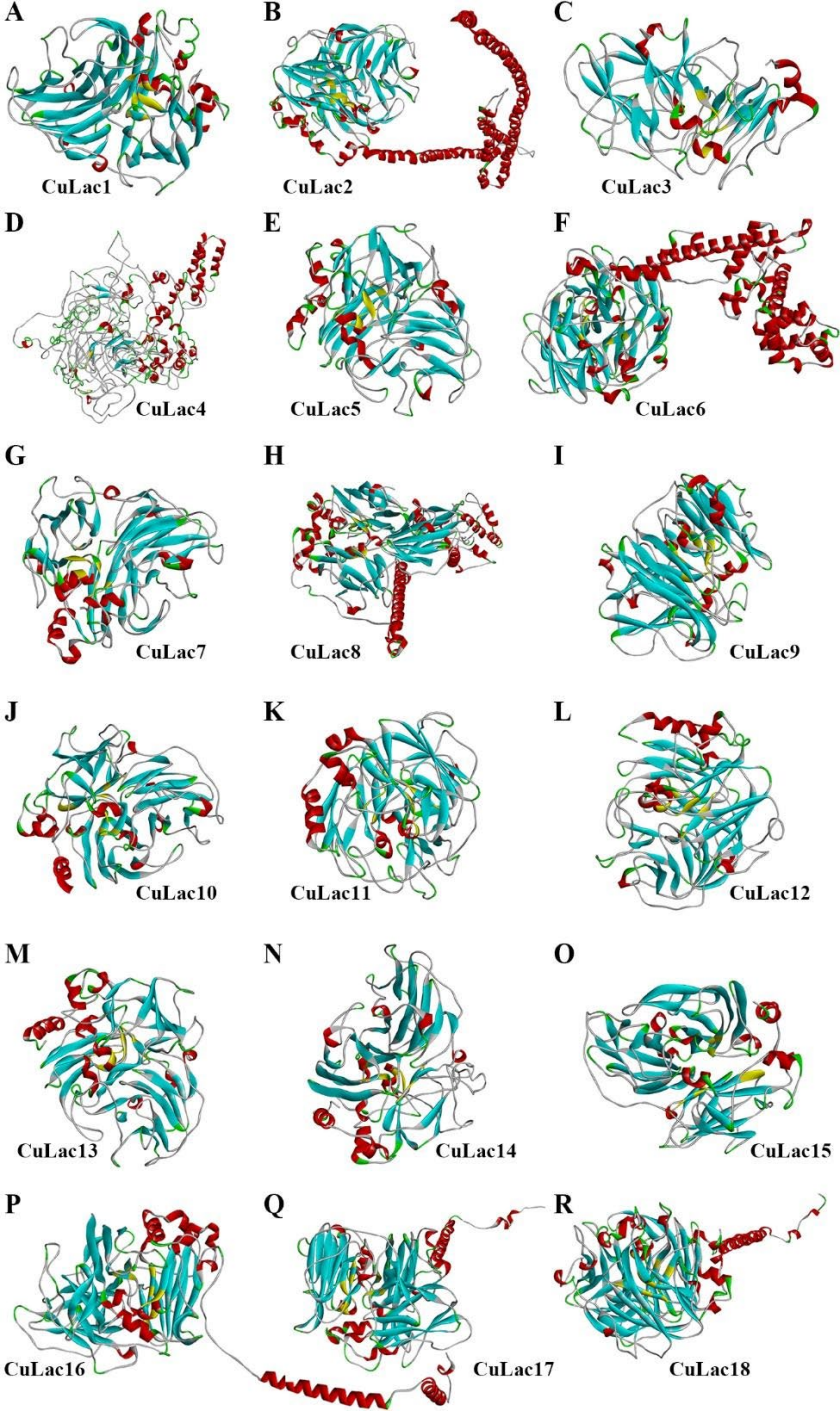
The three-dimensional structure of each CuLac protein built by Discovery Studio 2019 (V19.1.0)

**Fig. 7:**
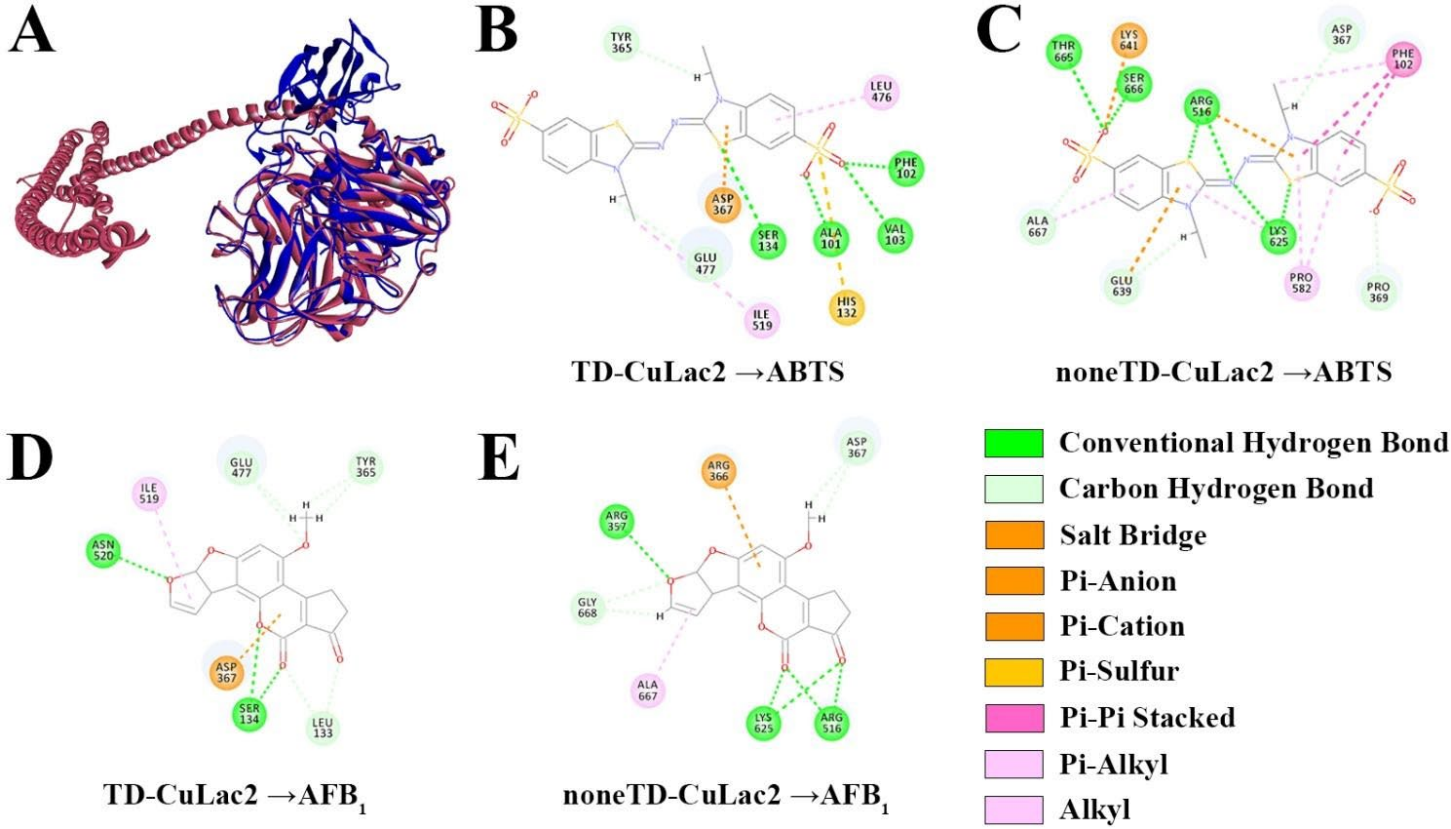
The structural and molecular-docking comparison of CuLac2 with or without full transmembrane domain (TD). **(A)** The structural alignment between TD-CuLac2 protein (red) and its TD-eliminated protein (noneTD-CuLac2, blue). **(B,C)** The interacting mode of TD-CuLac2 and noneTD-CuLac2 with substrate ABTS, respectively. **(D,E)** The interacting mode of TD-CuLac2 and noneTD-CuLac2 with substrate AFB_1_, respectively

Predicting the structure of laccase is crucial in uncovering its function and providing insight into how it catalyzes chemical reactions. In this study, we built 3D structure models of each CuLac isozyme using corresponding templates from Additional file 1: Table S5. Interestingly, a crystal structure of laccase from *Cerrena* sp. RSD1 (PDB ID: 5z1x) [48] was most frequently used as the basis for the models. Based on this structure, we built 3D structure models of CuLac1, 2, 9–15 (Fig. 6, Additional file 1: Table S5). Most CuLac isozymes had typical laccase structure, except for CuLac4 which had a clew-like structure (Fig. 6D). We also observed that eight CuLac isozymes had external sequences in N- or C-terminal with mostly α-helix structures, which were termed as transmembrane domain (TD) in CuLac2, 8, 16, 17, 18 or special sequence (SS) in CuLac4, 6 (Fig. 6B,D,F,H,P,Q,R). We built 3D structures of these special CuLac isozymes using both full-length sequence (TD/SS-CuLac) and TD/SS-eliminated sequence (noneTD/SS-CuLac) (Additional file 2: Fig. S6). It was worth mentioning that the amino acid sequence of SS-eliminated CuLac4 was successfully constructed into a normal laccase structure (Additional file 2: Fig. S6B). Except for CuLac4, the structural alignment between each pair of TD/SS-CuLac and noneTD/SS-CuLac showed similar structures constructed by the common sequences. For example, the common sequences (22–674 amino acids) of TD-CuLac2 and noneTD-CuLac2 constructed into similar secondary and tertiary patterns with slightly spatial mismatch (Fig. 7A).

However, these spatial mismatches might cause changes in the interacting mode between laccase and substrates. Therefore, we simulated the interacting modes of ABTS and AFB_1_ with both TD-CuLac2 and noneTD-CuLac2 structures. As shown in Fig. 7B,C, the ABTS-interacting mode towards the two structures was different both in interacting forces and interacting amino acid residues. Notably, the value of noneTD-CuLac2-ABTS docking energy (-3.34 kcal/mol) was higher than that scored (-4.32 kcal/mol) by TD-CuLac2-ABTS docking process (Additional file 1: Table S5), implying a higher binding affinity of noneTD-CuLac2 towards ABTS. Similarly, the interacting mode of AFB_1_ binding to noneTD-CuLac2 was altered (Fig. 7D,E), with noneTD-CuLac2 showing a higher docking energy score towards AFB_1_ (Additional file 1: Table S5).

We also performed molecular docking of ABTS and/or AFB_1_ with all CuLac structures. The CuLac family showed diverse interacting modes towards substrate ABTS or AFB_1_ (Additional file 2: Fig. S7, S8). Some CuLac isozymes (CuLac1–6, 8, 9, 12–14, 16, 17) showed higher docking-energy scores towards ABTS than that towards AFB_1_ (Additional file 1: Table S4). These results indicate that different members of the CuLac family have different substrate preferences and affinity to either ABTS or AFB_1_. Moreover, structures mimicked by the sequence of TD-CuLac16, 17 were unable to proceed with substrate docking for both ABTS and AFB_1_ (Additional file 2: Fig. S7T,V, Fig. S8T,V). These results suggest that the transmembrane domain has a more significant impact on CuLac16, 17 functions.

## Discussion

*Cerrena* is a genus of fungi that holds immense value in various fields, such as medicinal treatment and food processing, which mostly profits from its laccases production [8, 23]. Genome-wide sequencing has become a powerful technique to gain a deeper understanding of the *Cerrena* species. Although two *C. unicolor* species genomes have been published [22, 23], no characteristic and 3D-structural study of the laccase family in *C. unicolor* has been comprehensively reported. Moreover, these two genome sequences showed significant differences, including the numbers and identities of laccases. Therefore, conducting a genome-wide study of novel *Cerrena* species to explore laccases with diverse characteristics and 3D structures is still valuable.

In this study, we characterized *C. unicolor* 87613, which showed a maximal extracellular laccase activity of 415.66 U/mL at fermentation day 6, much earlier and higher productivity than those reported in other *Cerrena* species. For example, the peak value of *C. unicolor* CGMCC 5.1011 laccase activity was 121.7 U/mL at fermentation day 15 [26]. To better understand the genetic basis underlying its exceptional laccase production, we performed genome-wide sequencing and obtained a smaller-size genome (40.11 Mb) with more encoding genes (12,515) than *C. unicolor* SP02 (42.79 Mb, 12,277 predicted genes) [23]. These results indicate that *C. unicolor* 87613 genome is more intensively assembled with functional genes, which might contribute to its strain advantages, such as a predominance in laccase production. Indeed, we identified eighteen putative laccase genes in *C. unicolor* 87613, a number larger than those in known *Cerrena* species (having 5–10 laccases) [20, 22–24] and *Coprinopisis cinerea* (harboring the largest laccase gene family with 17 members) [17]. These findings indicate that *C. unicolor* 87613 might possess a more complex response strategy to environmental changes. The analysis of CuLac amino acid sequences revealed a molecular weight range of 47.89 to 141.41 kDa, while most of the fungal laccases ranged from 60 to 70 kDa [1]. These results implicate that there might be unique properties and/or functions of CuLacs. Among those, 16 out of 18 CuLacs contained a signal peptide, and 5 of them had transmembrane regions, suggesting that they were membrane laccases. Although laccases are usually secretory proteins, there also exists intracellular laccases [49, 50]. However, reports of membrane laccases in fungi are scarce. Furthermore, amino acid sequence analysis revealed multiple glycosylation sites in each CuLac, which might protect themselves against proteases [51]. Notably, correct glycosylation is required for heterologous expression of laccases in other hosts, such as *Pichia pastoris*, *Bacillus* sp., and *Aspergillus niger* [6]. Hence, identifying the CuLac glycosylation sites is crucial information for their efficient expression in heterologous hosts.

In terms of gene structure, we found that not all *CuLac* genes within the same Clade had similar exon-intron arrangements. This variability was uncommon in most known reports [13, 23], but has also been observed in the *Schizophyllum commune* 20R-7-F01 laccase family (Sc*LAC*) [52]. The diverse gene structures in *CuLac* genes suggest that their evolution and function might be varied. Nevertheless, amino acid sequences of all CuLacs exhibited the four conserved laccase signature sequences reported by Kumar et al. [32]. Interestingly, CuLac16–18 showed a change of cysteine to valine or threonine in the L2 signature sequence, which was often found in the ferroxidase amino acid sequence [17, 42]. Thus, CuLac16–18 might exhibit weak laccase activities but strong ferroxidase activities. This is supported by their phylogenetic classification into Clade III, which is separated from the other Clades. Furthermore, the tenth amino acid from the side of the cysteine residue (C) in L4 determined the redox potential of type I copper [53]. A larger hydrophobic group at this position contributed to a stronger redox potential. Therefore, laccases with phenylalanine (F) at this position showed the highest redox potential, followed by those with leucine (L), and laccase with methionine (M) at this position which showed the lowest redox potential [38, 39]. We observed that ten CuLacs contained phenylalanine residues at a related position, and eight proteins contained leucine residues at the same position. Most other Basidiomycete laccases had leucine and methionine at this position [54, 55]. These findings suggest that CuLacs are more promising laccases for industrial applications with relatively higher redox potential. Additionally, we performed a sequence analysis of substrate binding loops in CuLacs. Previous studies indicated that a cysteine (C), an aspartic acid (D), or a glutamic acid (E) was typically present in the β-hairpin loop B4–B5 of a fungal laccase [17]. However, some CuLac isozymes, such as CuLac2, 3, 9, did not contain these amino acids, suggesting weak catalytic capability as laccase. Moreover, the amino acid sequences of loop B4–B5 and B7–B8 in CuLac16–18 were significantly different from those in the other 15 isozymes but similar to those in ferroxidase-like PoLac5 in *P. ostreatus* [13]. These results infer the potential ferroxidase activities of CuLac16–18 as well.

*Cis*-acting elements are important for regulating spatiotemporal gene expression in different parts of the fruiting body and/or in response to different environmental cues [56]. We scanned 1,500 bp upstream of the start codons of *CuLac* genes and identified nine important *cis*-acting elements in their promoter regions. Among those, nitrogen binding site (NIT2) was the most frequently occurring element, which was similar to *Pleurotus ostreatus* [13]. These results indicate that changes in nitrogen source might significantly impact on the transcription of fungal laccase genes, as reflected in the number of NIT2 elements in *CuLac* gene family. Copper ion binding site (ACE1) was the second-largest element, indicating that the transcription of *CuLac* could be induced by additional copper ions [57–59]. Furthermore, CreA-binding site sequences were largely found in the promoter regions of 13 *CuLac* genes, implicating that the changes in carbon source might also influence the transcription levels of laccase genes. The existence of antioxidant response element (ARE) in the promoter regions of *CuLac6*, *9*, *10*, *13*, *15*–*18* suggests that they might play responsive roles to oxidative cues [60]. Other *cis*-acting elements, including metal responsive element (MRE), xenobiotic responsive element (XRE) and stress response element (STRE), were also identified. The complex pattern of *cis*-acting elements suggests that the regulation of *CuLac* gene expression is complicated and multi-faceted. In this study, we also elucidated the expression patterns of each *CuLac* gene under different conditions. Substitution of glucose with fructose reduced the expression of most *CuLac* genes, indicating that fructose is not the preferred carbon source for their expression. However, the insignificant changes of *CuLac1*, *4*, *15*, *16* expressions maintained the crude laccase activities. Besides, most *CuLac* genes were repressed by low nitrogen concentration, consistent with the presence of NIT2. However, low nitrogen concentration also increased the expression levels of *CuLac12*, *17/18*, a contradictory phenomenon previously observed in *C. unicolor* FCL139 [20]. In addition, most *CuLac* genes were upregulated in the presence of high copper ions or H_2_O_2_ concentration, but there were exceptions. Particularly, we identified *CuLac8* and *CuLac2* as the strongest contributors to the elevation of intracellular laccase activities in response to copper ions and H_2_O_2_, respectively. Meanwhile, *CuLac12* and *CuLac13* were found to strongly contribute to the increase in extracellular laccase activities in response to copper ions and H_2_O_2_, respectively. Interestingly, in many *Cerrena* species, there commonly existed a predominant laccase isozyme contributing to the total laccase activity [11, 12]. Our study further deduced that the predominant laccase isozyme might be shifted under different stimuli.

Previous studies on the laccase gene family lacked a 3D-structure analysis and molecular docking with substrates. Herein, we built the 3D structures of all 18 CuLac isozymes and found that CuLac2, 4, 6, 8, 16, 17, 18 had an additional transmembrane domain (TD) or special sequence (SS), respectively. We discovered that the presence or absence of these domains affects the laccase’s affinity to substrates, which might be attributed to their influences on the interacting bonds and amino acid residues between laccase and substrates. For example, TD-eliminated CuLac2 showed more conventional hydrogen bonds and carbon-hydrogen bonds toward ABTS than those in TD-CuLac2. These deductions are also referred to AFB_1_. As compared to laccases from *C. unicolor* strain 6884 (-33.99 and − 34.53 kcal/mol) [29], we also found that most CuLac isozymes displayed higher affinities to AFB_1_ (from − 31.69 to -19.05 kcal/mol), indicating their potential for AFB_1_ detoxification. It is worth mentioning that substrates with a similar aromatic structure to ABTS and/or AFB_1_ might produce similar results as mentioned above. However, substrates with different structures still require further analysis to determine their outcomes.

## Conclusions

In this study, we conducted a genome-wide study of laccase gene family in *C. unicolor* 87613, a strain known for early and high-laccase production. Our research identified eighteen putative laccase genes (*CuLac*s), and comprehensively explored the characteristics of their genes. In addition, we unveiled their *cis*-acting elements and their transcription patterns under different conditions. Furthermore, we built 3D structure models of each CuLac for molecular docking to ABTS and AFB_1_, respectively. Our findings provide detailed and valuable information about the *C. unicolor* 87613 laccase family, contributing to a deeper understanding of *CuLac* genes and their potential for more effective industrial application.

### Electronic supplementary material

Below is the link to the electronic supplementary material.

Supplementary Material 1

Supplementary Material 2

## Data Availability

The assembled sequences data from the *Cerrena unicolor* 87613 genome are available at Sequence Read Archive (SRA) GenBank database, deposited under the Accession Number No. SRR23097119 (BioProject: PRJNA924695). Putative laccase genes were also deposited in GenBank under the Accession Numbers No. OQ863206-OQ863223 for *CuLac1*-*CuLac18*, respectively.
