# Supplementary material for: Genome-wide study of Cerrena unicolor 87613 laccase gene family and their mode prediction in association with substrate oxidation
Source: BMC Genomics. 2023 Aug 30;24:504. doi: 10.1186/s12864-023-09606-9 (PMC10466755; doi:10.1186/s12864-023-09606-9)
Supplement: Supplementary file 1 — Supplementary Material 1 [file 12864_2023_9606_MOESM1_ESM.docx]

**Table S1 The characteristics of *C. unicolor* 87613 genome**

| ***C. unicolor* Species** | **Raw Data**  **(Mb)** | **Filtered Reads**  **(%)** | **Clean Data**  **(Mb)** | **Clean Data GC**  **(%)** | **Clean Data Q20**  **(%)** | **Clean Data Q30**  **(%)** | **Genome Size**  **(Mb)** | **Heterozygous Rate**  **(%)** | **Repeat Rate**  **(%)** | **Total Predicted Genes** | **Protein-Coding Genes** |
| --- | --- | --- | --- | --- | --- | --- | --- | --- | --- | --- | --- |
| sp. 87613 | 6,174 | 21.71 | 4,834 | 46.64 | 98.11 | 94.36 | 40.11 | 0.94 | 26.29 | 26,799 | 12,515 |

Raw data: raw data volume;

Filtered reads: the proportion of filtered data in the original data;

Clean data: the effective amount of filtered data;

Clean data GC: average GC content of effective data;

Clean data Q20: Q20 value of valid data;

Clean data Q30: Q30 value of valid data;

Heterozygous rate: estimated genomic heterozygosity ratio;

Repeat rate: estimated genome repeat ratio

**Table S2 Sequences of 15 conserved motifs.**

| **Motif No.** | **Sequence (N-terminal → C-terminal)** |
| --- | --- |
| Motif 1 | GTFWYHSHLSTQYCDGLRGAFVVYDPNDPHKDLYDVDDESTVITLADWYH |
| Motif 2 | TDASMLKSTSIHWHGFFQKGTNWADGPAFVNQCPITTGNSFLYDFTVPDQ |
| Motif 3 | DNVTIRFVTDNPGPWFLHCHIDWHLEAGFAVVFAEGINZTA |
| Motif 4 | VINVEAGKRYRFRLVSISCDPNFVFSIDNHBMTVIEVDGVN |
| Motif 5 | LTVDSIQIFAGQRYSFVLNANQPVDNYWIRANPNLGTTGF |
| Motif 6 | AYAAIGPVADLHIVBADIAPDGFTRPAVLAGGTFPGPLITGNKGDNFKLN |
| Motif 7 | INSAILRYKGAPVAEPTTSQTPSTKPLLETBLHPLVSTPVPGLPVPGGAD |
| Motif 8 | VVGGPHPFHLHGHNFHVVRSAGQDTYNYDDPIVRDVVNIGA |
| Motif 9 | GASFTPPSVPVLLQILSGTTNAQDLLPSGSVIELPLGKTVE |
| Motif 10 | ARTVVGVAIPKTTLINGLGRSHBGPADAE |
| Motif 11 | AANPTPAAWDNLCPKYBAL |
| Motif 12 | HNLILGFNAGRFTIN |
| Motif 13 | YWIRTQIQTDMFTYDVPGQNTDIRGVLRYSNAQDPPMLPVATMDPGSGKK |
| Motif 14 | TDPAPKGFPTWDQDLVIQVNDLYHTFNPFVEGDGVDSLLESMEHLGMNQF |
| Motif 15 | RFLAFMNGTSWEPLNGTSTLLAVQHAGGSEFAPSGGSLQDYSQFLITENS |

**Table S3 The conserved sequences of each *cis*-element.**

| ***Cis*-element** | **Conserved sequences (5’→ 3’)** |
| --- | --- |
| TATA box | TATAWAW |
| CAAT box | GGNCAATCT |
| GC box | GCCGCC or GGGCGGGG |
| Copper ion binding site (ACE1) | HWHNNGCTGD or NTNNHGCTGN |
| Antioxidant response element (ARE) | TGACNNNGC |
| cAMP-mediated glucose repression response element (CreA) | SYGGRG |
| Nitrogen repression response element (NIT2) | TATCDH |
| Metal responsive element (MRE) | TGCRCNC |
| Xenobiotic responsive element (XRE) | TNGCGTG |
| Stress response element (STRE) | CCCCT |

W stands for A or T; N stands for C or T; H stands for A, C or T; D stands for A, G or T; S stands for C or G; Y stands for C or T; R stands for A or G.

**Table S4 Sequences of primers for qRT-PCR.**

| **Primer pairs** | **Sequences (5’ → 3’)** | **Purpose** |
| --- | --- | --- |
| *Lac1*-F / *Lac1*-R | TTACCTTCTCCATTGATG / GTCCAGTGTTGTTGTATT | qRT-PCR of *CuLac1* |
| *Lac2*-F / *Lac2*-R | CCGTTGATGCTGCGAATG / TTGTAAGTAAGGAGTGTGAGGTAG | qRT-PCR of *CuLac2* |
| *Lac3*-F / *Lac3*-R | CATCTCTTGGTCTTGTCT / TACTCTGTAGGCATTGTG | qRT-PCR of *CuLac3* |
| *Lac4*-F / *Lac4*-R | AGGTCAACGATATTCATT / GTAATATAGCGGAGTTCA | qRT-PCR of *CuLac4* |
| *Lac5*-F / *Lac5*-R | CCTGGAGCACTTATCACT / ACATACTGGCATCGGTAA | qRT-PCR of *CuLac5* |
| *Lac6*-F / *Lac6*-R | TAGGATGGATAGATGTGAAC / CAATATGGCGAGACTGTA | qRT-PCR of *CuLac6* |
| *Lac7*-F / *Lac7*-R | GGTCCTCTTGTTGTGTAT / GGTGATAACGGTAGTCTC | qRT-PCR of *CuLac7* |
| *Lac8*-F / *Lac8*-R | TATGACCATTATTGAAGTTG / ACGAATCCAGTAGTTATC | qRT-PCR of *CuLac8* |
| *Lac9*-F / *Lac9*-R | TTGAAGTGGATAGTGTTA / TTGATACCATTGTTGAAG | qRT-PCR of *CuLac9* |
| *Lac10*-F / *Lac10*-R | CTCTGTCATTAGCGTTCA / TCATATTATGGTTGTCAATC | qRT-PCR of *CuLac10* |
| *Lac11*-F / *Lac11*-R | TCGTGCTCTTAGTGCTTA / TCCGTGGAGATATTGGTATT | qRT-PCR of *CuLac11* |
| *Lac12*-F / *Lac12*-R | GAATCCACTGTTATCACCTT / TTGCGACCTAATCCATTG | qRT-PCR of *CuLac12* |
| *Lac13*-F / *Lac13*-R | CGAAGTTGATGGTGTTAA / ACGAATCCAGTAATTGTC | qRT-PCR of *CuLac13* |
| *Lac14*-F / *Lac14*-R | TTGCTGGTGGTATCAATTC / TGGTCTGCGAAGTAGTAG | qRT-PCR of *CuLac14* |
| *Lac15*-F / *Lac15*-R | CATTGAAGTTGATGGTGTT / ACGAATCCAGTAGTTGTC | qRT-PCR of *CuLac15* |
| *Lac16*-F / *Lac16*-R | AACACCAATATGACGAAGAAT / GGCAACACTGATGAACTG | qRT-PCR of *CuLac16* |
| *Lac17/18*-F / *Lac17/18*-R | GTCCTCGTCCATACCAAT / ATAAGTGAACATATCCGTCTG | qRT-PCR of *CuLac17* and *CuLac18* |
| 18S-F / 18S-R | AGACGGAAGTTTGAGGCA / CTTCCGGCCAAGGTGAA | Internal standard |

**Table S5 Information of homology models of each *C.unicolor* laccases and their molecular docking with ABTS and/or AFB_1_**

| **Term** | **Amino acid chain** | **CHARMm Energy (kcal/mol)** | **Template** | **Identity** | **-CDOCKER_Energy to ABTS (kcal/mol)** | **-CDOCKER_Energy to AFB_1_ (kcal/mol)** |
| --- | --- | --- | --- | --- | --- | --- |
| CuLac1 | 22-526 | -31,220.34 | 5z1x | 0.713 | -5.08 | -24.40 |
| TD-CuLac2 | 22-764 | -42,911.13 | 5z1x | 0.709 | -4.32 | -25.51 |
| noneTD-CuLac2 | 22-674 | -41,157.88 | 5z1x | 0.710 | -3.34 | -19.05 |
| CuLac3 | 32-430 | -23,261.58 | 5z22 | 0.580 | -6.15 | -24.75 |
| SS-CuLac4 | 20-1304 | -76,411.77 | 3h7n | 0.086 | -5.42 | -30.21 |
| noneSS-CuLac4 | 20-520 | -28,957.59 | 5e9n | 0.680 | -1.71 | -20.75 |
| CuLac5 | 22-526 | -30,821.57 | 5z1x | 0.731 | -13.73 | -29.07 |
| SS-CuLac6 | 21-815 | -48,779.87 | 5e9n | 0.650 | -5.68 | -24.39 |
| noneSS-CuLac6 | 21-530 | -30,880.83 | 5e9n | 0.650 | -18.36 | -29.19 |
| CuLac7 | 21-510 | -29,844.60 | 5z1x | 0.640 | -55.33 | -25.34 |
| TD-CuLac8 | 22-844 | -47,392.83 | 3kw7 | 0.690 | -12.15 | -28.51 |
| noneTD-CuLac8 | 22-625 | -33,497.96 | 1gyc | 0.690 | -19.24 | -28.90 |
| CuLac9 | 21-483 | -27,635.53 | 5z1x | 0.746 | -18.57 | -25.02 |
| CuLac10 | 22-516 | -29,051.08 | 5z1x | 0.794 | -27.23 | -27.19 |
| CuLac11 | 22-516 | -30,314.57 | 5z1x | 0.758 | -75.20 | -23.08 |
| CuLac12 | 22-518 | -30,848.43 | 5z1x | 0.802 | -12.34 | -31.69 |
| CuLac13 | 22-516 | -30,937.45 | 5z1x | 0.859 | -13.75 | -28.19 |
| CuLac14 | 22-537 | -31,013.72 | 5z1x | 0.834 | -17.56 | -25.35 |
| CuLac15 | 22-516 | -28,837.61 | 5z1x | 0.881 | -135.21 | -25.40 |
| TD-CuLac16 | 18-627 | -35,395.89 | 1zpu | 0.370 | Failed | Failed |
| noneTD-CuLac16 | 18-553 | -33,117.75 | 1zpu | 0.390 | -24.63 | -26.34 |
| TD-CuLac17 | 1-597 | -34,956.21 | 5lm8 | 0.305 | Failed | Failed |
| noneTD-CuLac17 | 51-597 | -33,124.09 | 4jhu | 0.330 | -20.61 | -31.38 |
| TD-CuLac18 | 1-612 | -35,808.90 | 5lm8 | 0.310 | -148.169 | -62.86 |
| noneTD- CuLac18 | 51-612 | -34,605.86 | 4jhu | 0.320 | -188.27 | -25.91 |
