# Supplementary material for: Genome-wide study of Cerrena unicolor 87613 laccase gene family and their mode prediction in association with substrate oxidation
Source: BMC Genomics. 2023 Aug 30;24:504. doi: 10.1186/s12864-023-09606-9 (PMC10466755; doi:10.1186/s12864-023-09606-9)
Supplement: Supplementary file 2 — Supplementary Material 2 [file 12864_2023_9606_MOESM2_ESM.docx]

**Supplementary figures**

**
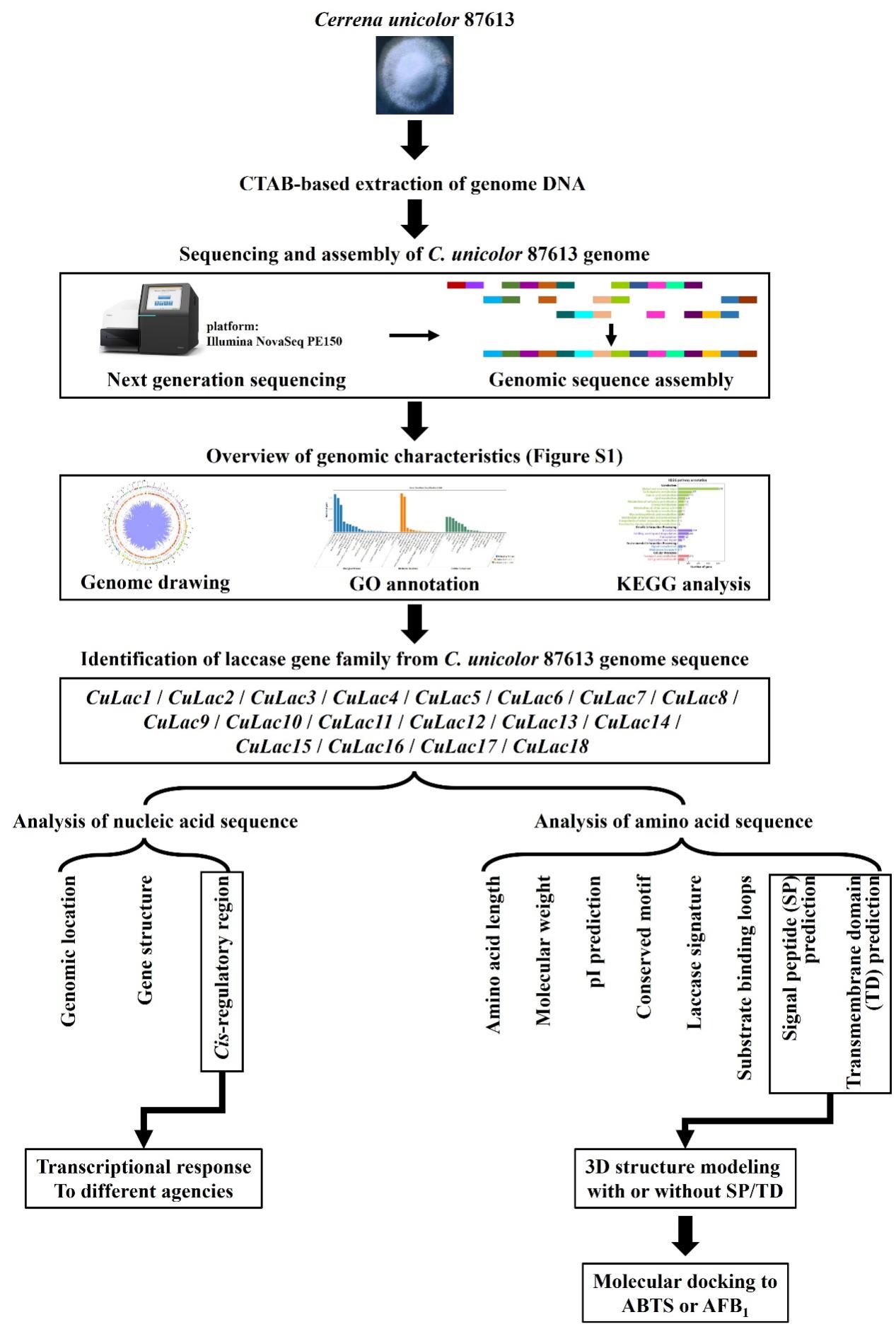
**

**Fig. S1** Illustration of this experimental flow.

**
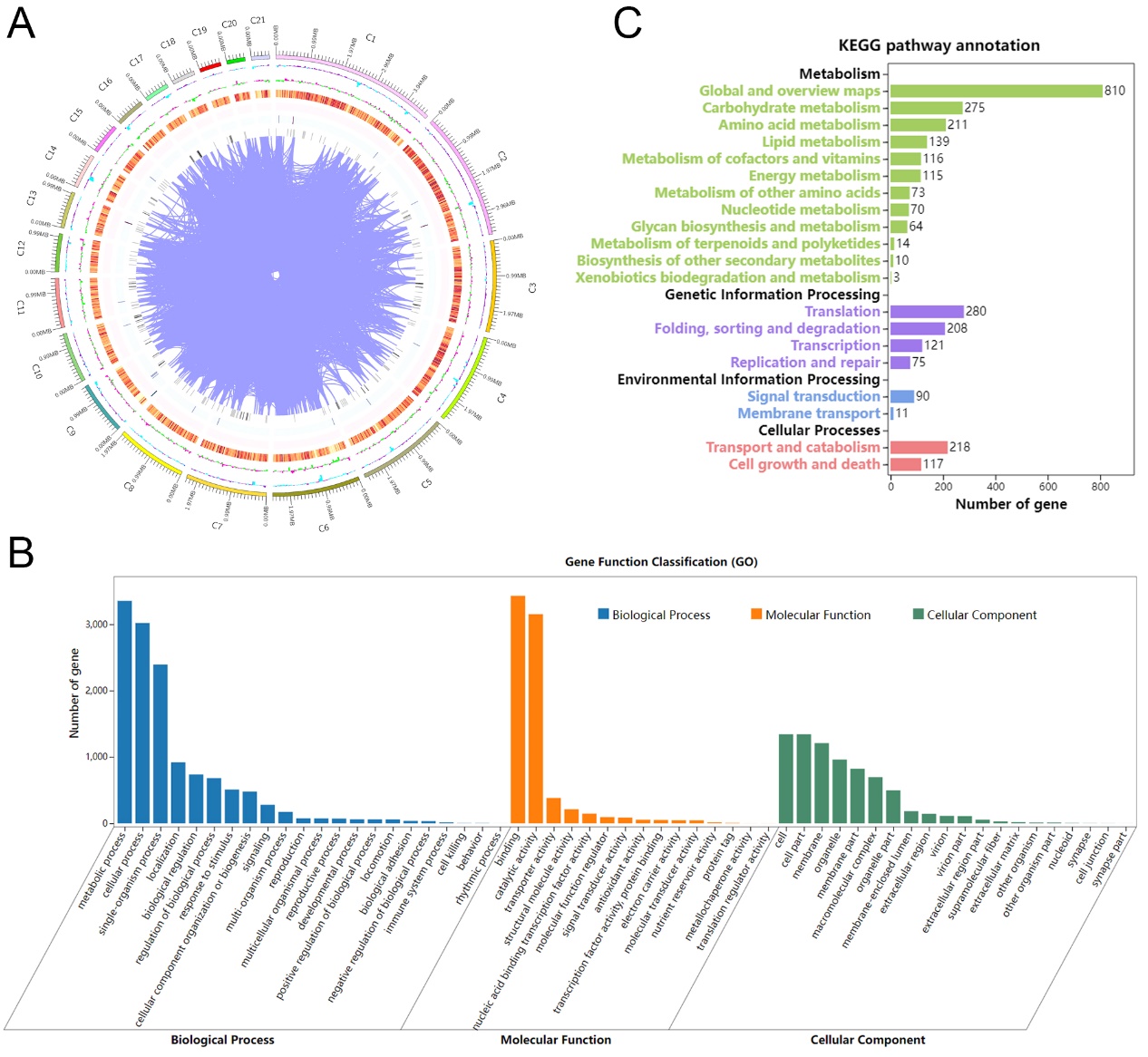
**

**Fig. S2** Bioinformatic analysis of *C. unicolor* 87613 genome data. (**A**) Circular genome map of *C. unicolor* 87613. (**B**) The Gene Ontology (GO) function annotation of *C. unicolor* 87613. (**C**) Clusters of the KEGG functional classification of *C. unicolor* 87613 genes.


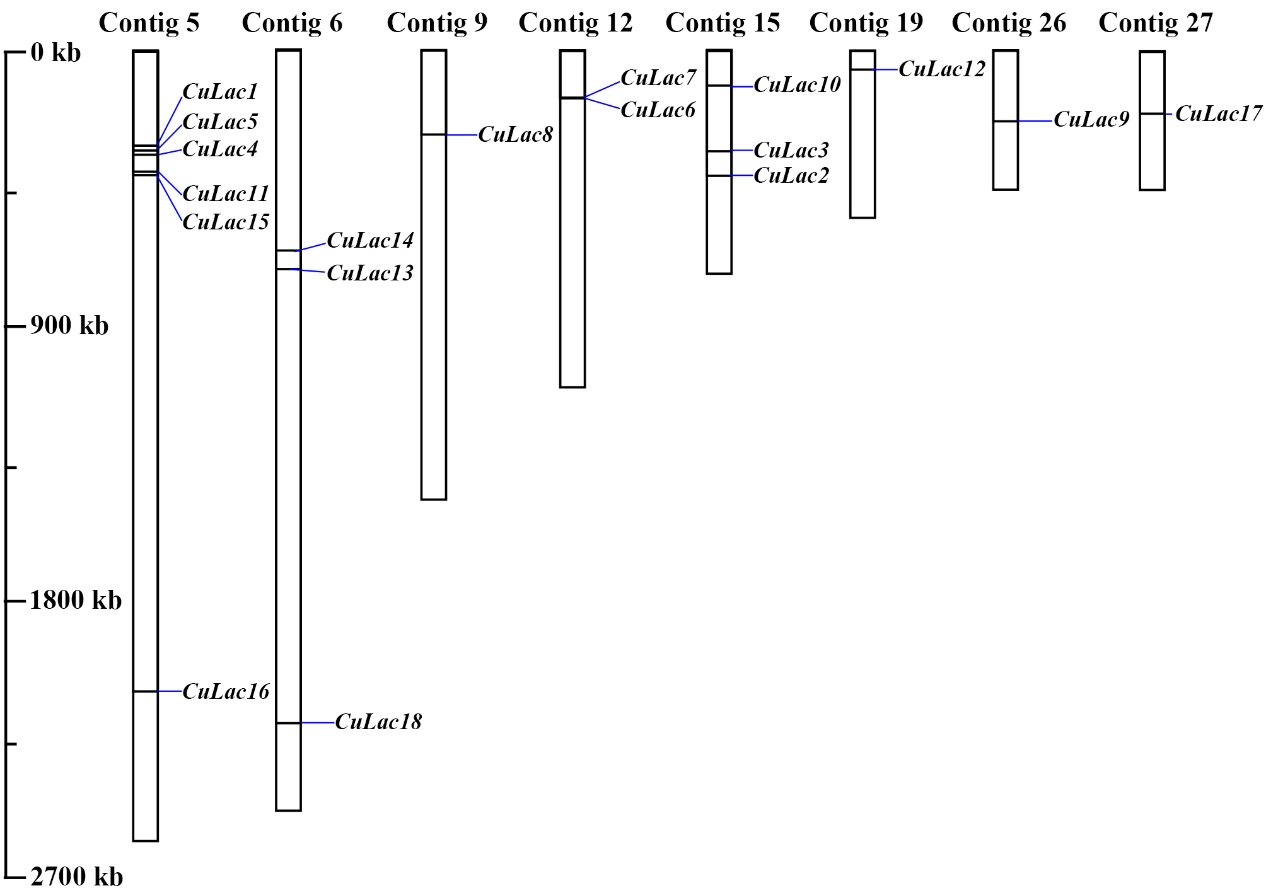


**Fig. S3** Physical map of the C. unicolor 87613 laccase genes. The localization of each *CuLac* gene to contigs (the assembled units of *C. unicolor* 87613 genome).


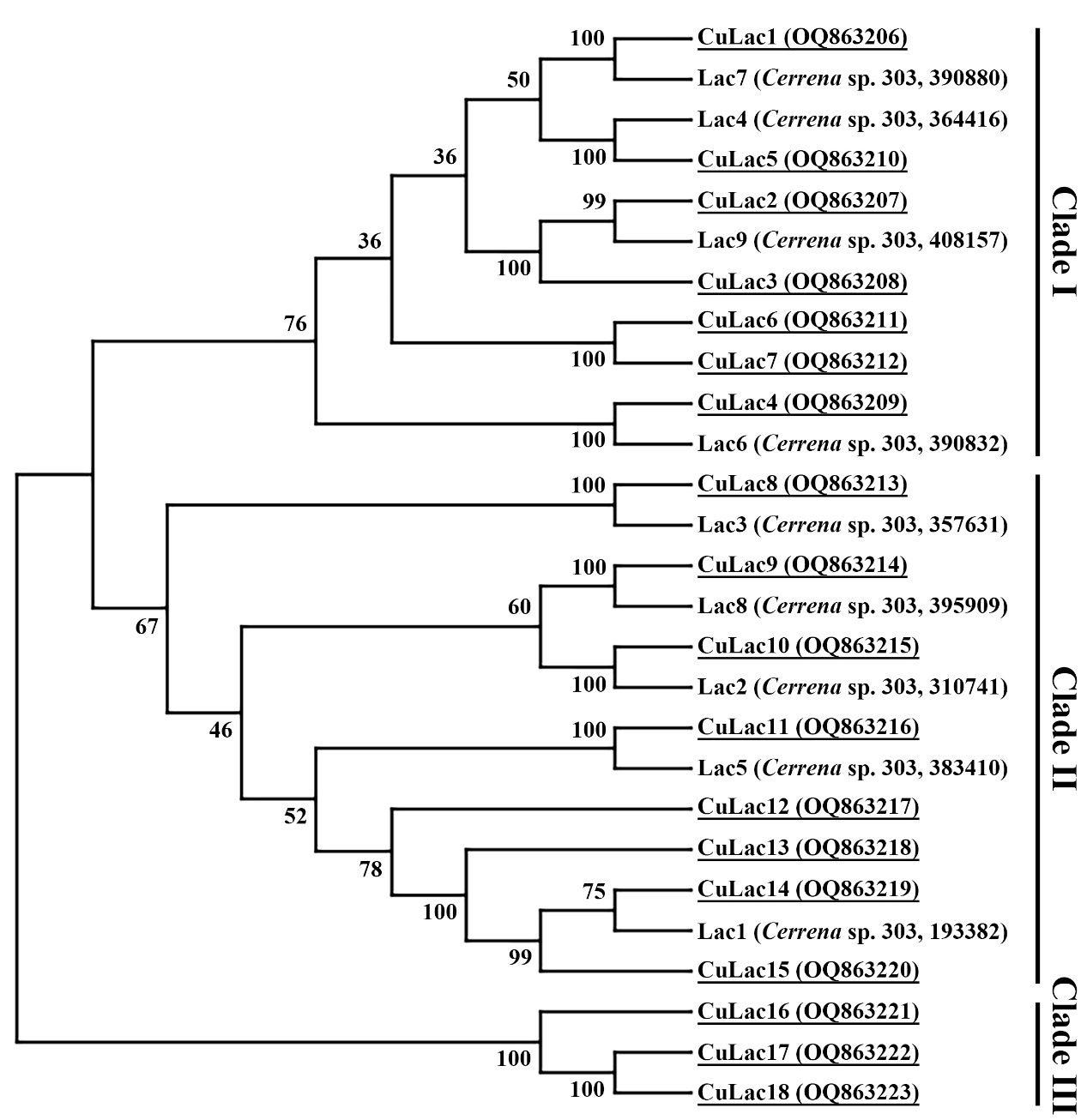


**Fig. S4** Phylogenetic analysis of 18 laccase genes (*CuLac1*–*18*) in *C. unicolor* 87613 with 9 laccase genes (*Lac1*–*9*) in *Cerrena* sp. 303. The accession IDs of each *CuLac* gene were available in NCBI Genbank, and the accession IDs of each laccase in *Cerrena* sp. 303 were available in JGI database.


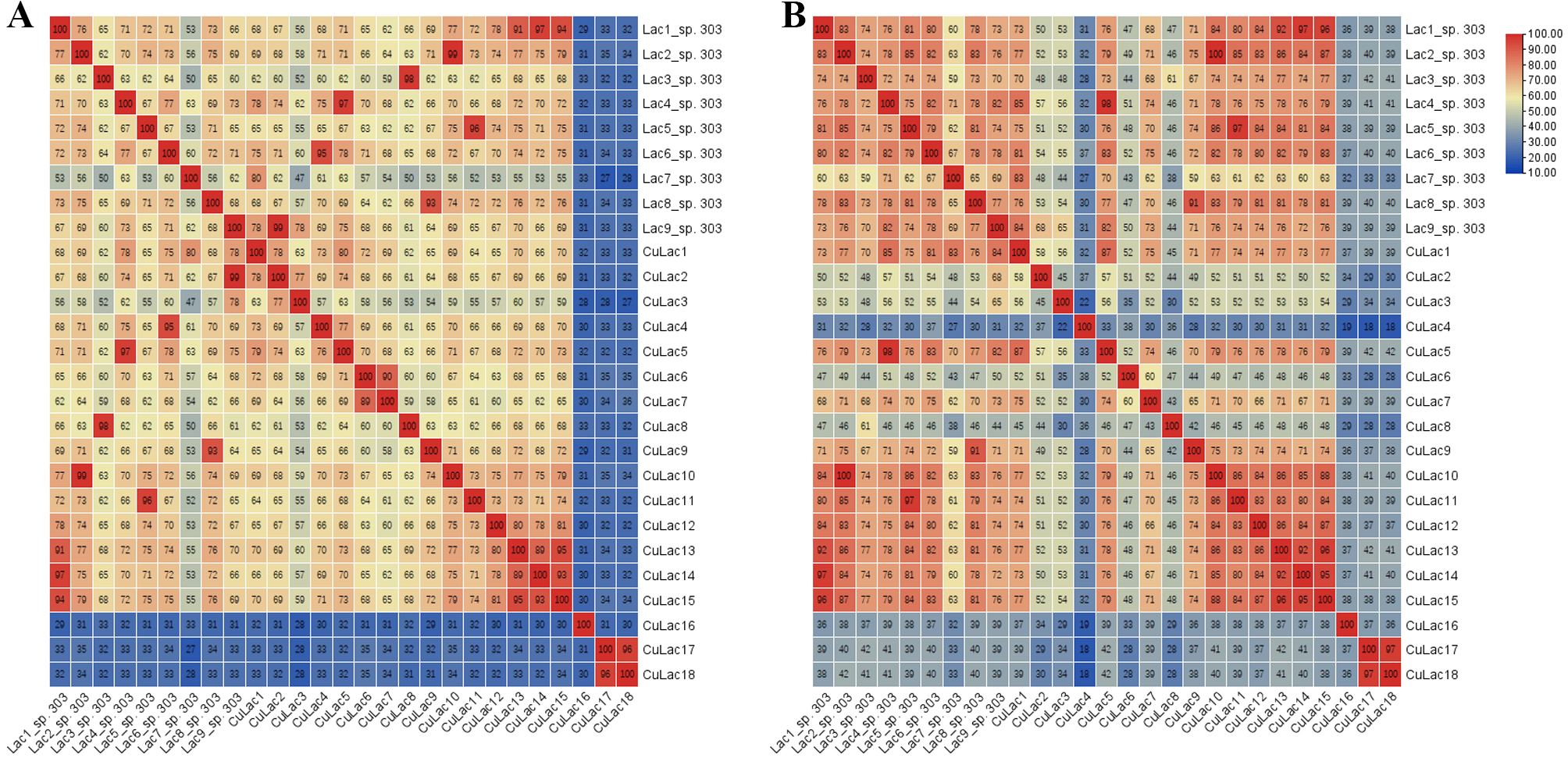


**Fig. S5** Pairwise similarity and identity matrix among CuLac proteins and laccases in *Cerrena* sp. 303. (**A**) The protein pairwise similarity matrix. (**B**) The protein pairwise identity matrix.


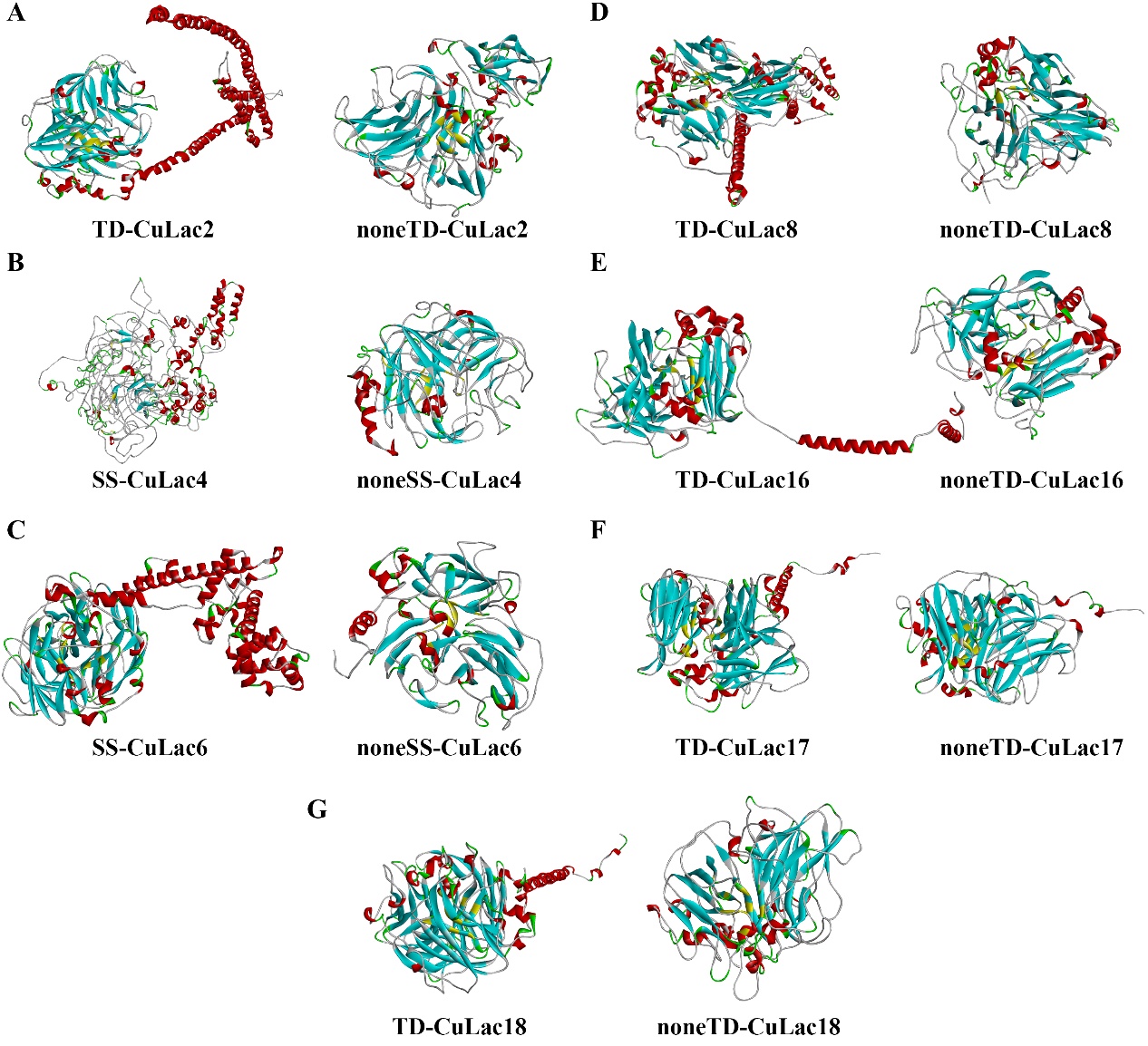


**Fig. S6** The 3D-structural comparison of CuLac proteins with or without extra transmembrane domain (TD) or special sequence (SS). (**A**-**G**) The comparison of different structures of CuLac2, 4, 6, 8, 16, 17, 18, respectively.


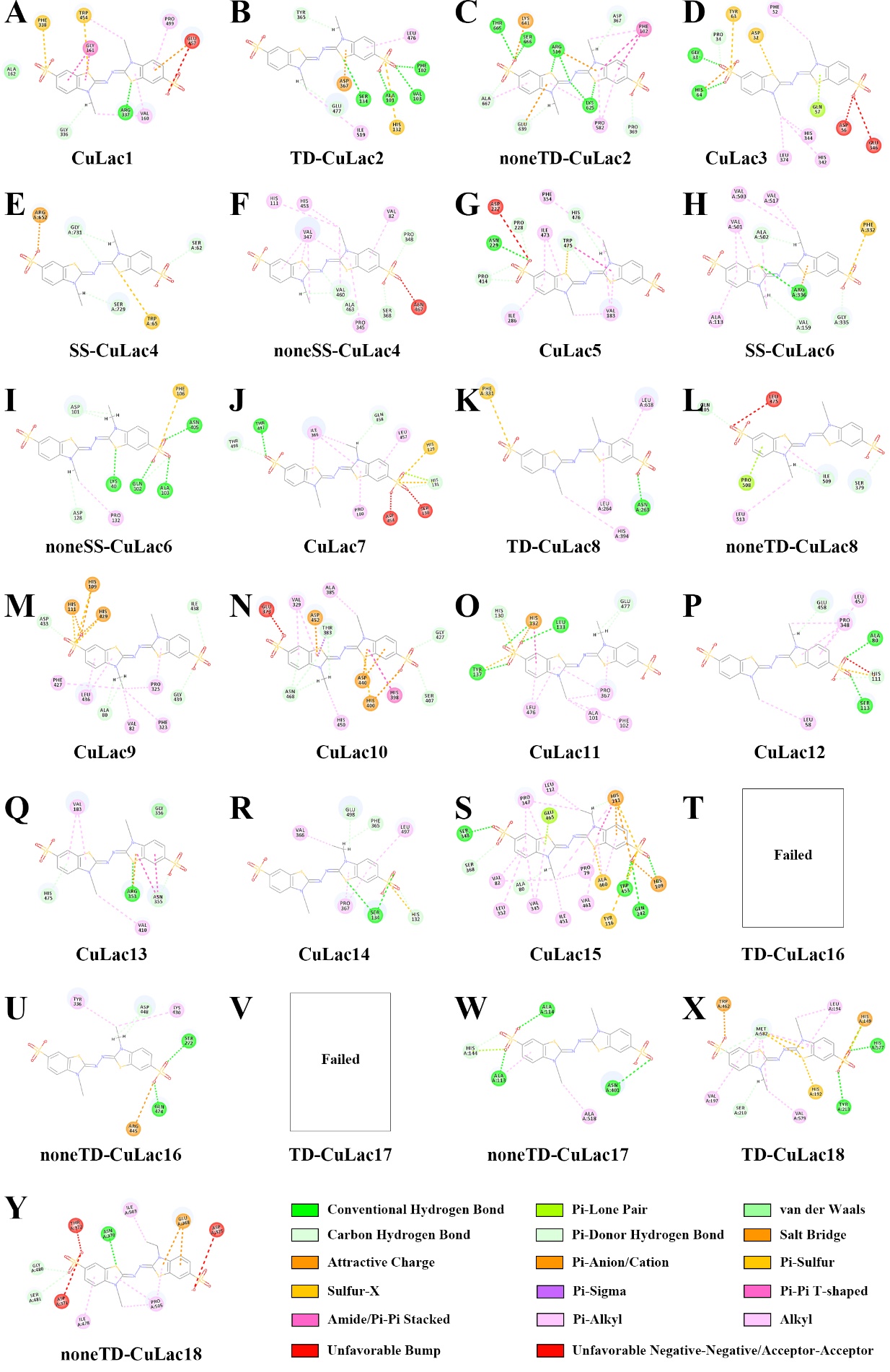


**Fig. S7** The molecular docking modes of each CuLac structure with substrate ABTS. (**A**-**Y**) The presentation of interacting residues and bonds between CuLac proteins and ABTS.


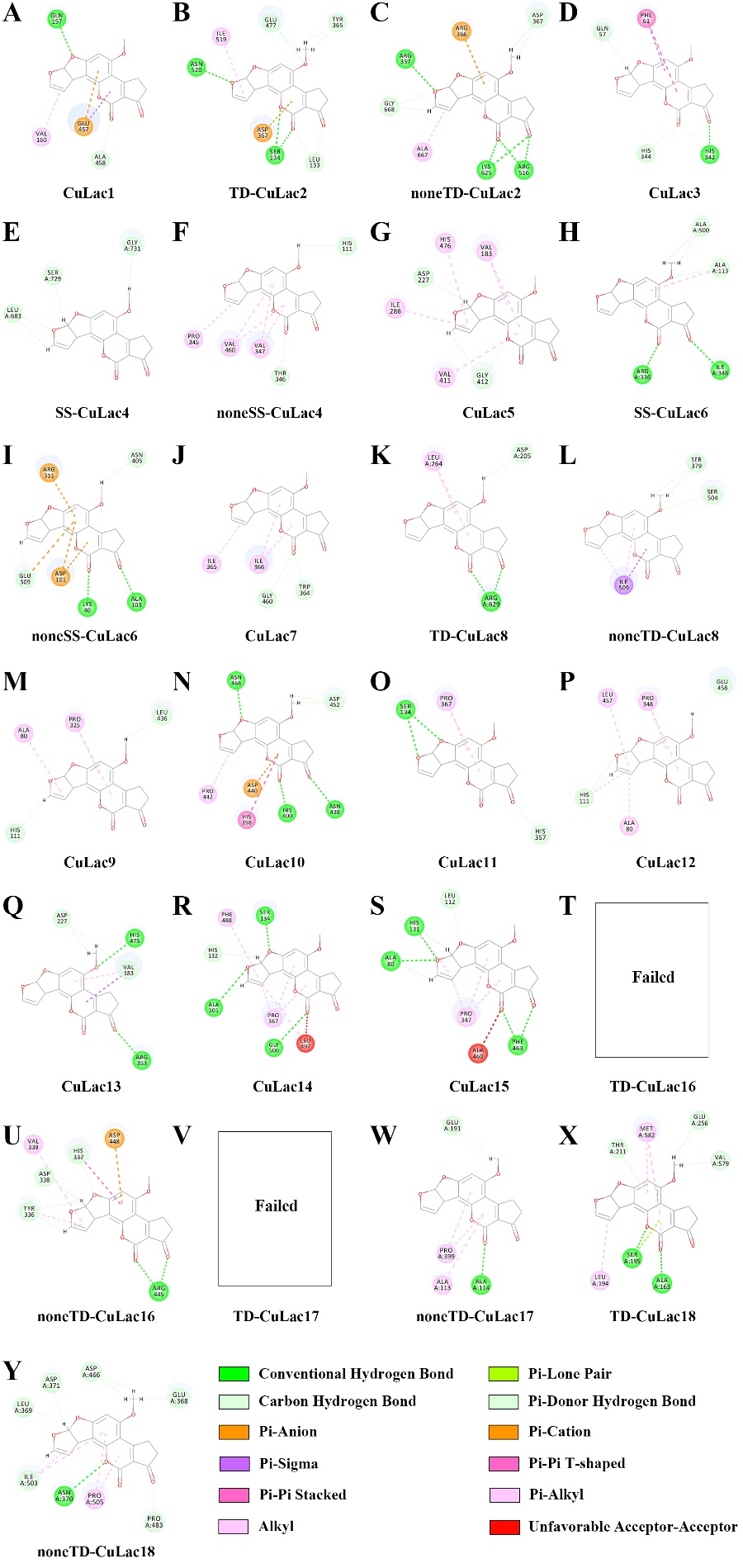


**Fig. S8** The molecular docking modes of each CuLac structure with substrate AFB_1_. (**A**-**Y**) The presentation of interacting residues and bonds between CuLac proteins and AFB_1_.
